# B cells maintain the homeostasis of splenic marginal zone antigen-presenting cells to promote the antiviral CD8^+^ T-cell response

**DOI:** 10.1038/s41423-026-01392-0

**Published:** 2026-02-24

**Authors:** Xinyuan Liu, Filiz Demircik, Mariia Antipova, Emmanouil Stylianakis, Matthias Klein, David Bejarano, Abdelrahman Elwy, Anna Ebering, Michaela Blanfeld, Katlynn Carter, Lisa Johann, David C. Uhlfelder, Elisa Blickberndt, Yao Chen, Hans Christian Probst, Nadine Hövelmeyer, Tobias Bopp, Ramon Arens, Lukas Bunse, Joke M. M. den Haan, Jennifer L. Gommerman, Esther von Stebut, Björn E. Clausen, Andreas Schlitzer, Karl S. Lang, Bing Su, Ronald A. Backer, Niels A. Lemmermann, Ari Waisman

**Affiliations:** 1 Institute for Molecular Medicine, University Medical Center of the Johannes Gutenberg -University Mainz, Mainz, Germany; 2 Institute for Immunology, University Medical Center of the Johannes Gutenberg- University Mainz, Mainz, Germany; 3 Research Center for Immunotherapy (FZI), University Medical Center of the Johannes Gutenberg- University Mainz, Mainz, Germany; 4 Quantitative Systems Biology, Life & Medical Sciences Institute, University of Bonn, Bonn, Germany; 5 Institute of Immunology, Medical Faculty, University Duisburg-Essen, Essen, Germany; 6 Shanghai Institute of Immunology, Department of Immunology and Microbiology, Shanghai Jiao Tong University School of Medicine, Shanghai, China; 7 Department of Immunology, Leiden University Medical Center, Leiden, The Netherlands; 8 DKTK Clinical Cooperation Unit (CCU) Neuroimmunology and Brain Tumor Immunology, German Cancer Research Center (DKFZ), Heidelberg, Germany; 9 Department of Neurology, Medical Faculty Mannheim, Mannheim Center for Translational Neuroscience (MCTN), Heidelberg University, Heidelberg, Germany; 10 Department of Molecular Cell Biology and Immunology, Cancer Center Amsterdam, Amsterdam Institute for Infection and Immunity, Amsterdam UMC, Vrije Universiteit Amsterdam, Amsterdam, The Netherlands; 11 Department of Immunology, University of Toronto, ON, Toronto, Canada; 12 Department of Dermatology, Medical Faculty, University of Cologne, Cologne, Germany; 13 Institute for Virology, University Medical Center of the Johannes Gutenberg- University Mainz, Mainz, Germany; 14 Institute for Virology, Medical Faculty, University Bonn, Bonn, Germany

**Keywords:** B cells, splenic marginal zone antigen-presenting cells, anti-viral CD8^+^ T-cell response, Infection, Antigen processing and presentation

## Abstract

Natural killer and CD8^+^ T cells are critical in the elimination of blood-borne viruses such as cytomegalovirus (CMV); however, the role of B cells in this process is less clear. Here, using a murine CMV (MCMV) infection model, we demonstrated that B-cell-deficient mice mounted a weaker primary virus-specific CD8^+^ T-cell response than their wild-type counterparts did, which was associated with increased viral transcription. Notably, we found that the contribution of B cells to the CD8^+^ T-cell-mediated antiviral response was not associated with their ability to generate antibodies but with their ability to sustain Langerin^+^ type 1 conventional dendritic cells (cDC1s), a dendritic cell (DC) subset known for being involved in viral and bacterial clearance in the marginal zone of the spleen. Furthermore, we found that the presence of Langerin^+^ cDC1s is dependent on B cells expressing lymphotoxin (LTβ) to maintain CD169^+^ marginal metallophilic macrophages (MMMs). We further discovered, via ligand‒receptor interaction analyses, that the communication between MMMs and Langerin^+^ cDC1s was mediated via the VCAM1–ITGA4/ITGB1 interaction. Thus, our data reveal that B cells regulate the development of MMMs in the spleen via LTβ expression and consequently sustain Langerin^+^ cDC1 homeostasis for effective initiation of an antiviral CD8^+^ T-cell response. Overall, our study offers a new perspective on how B cells maintain the homeostasis of antigen-presenting cells in the splenic marginal zone and thus indirectly affect the virus-specific CD8^+^ T-cell response, which could be extended to other infectious and autoimmune diseases as well as tumors.

## Introduction

Cytomegalovirus (CMV), which belongs to the β subfamily of herpesviruses, establishes lifelong latent infection in humans [1]. Because CMV is strictly species specific, murine CMV (MCMV) serves as a well-established animal model for human CMV (HCMV) pathogenesis and immune control [2, 3]. Although HCMV does not typically cause symptomatic disease in immunocompetent individuals, it is often reactivated in immunocompromised bone marrow (BM) transplant recipients, causing severe disease [4, 5]. In these patients, the clearance of CMV infection relies on the efficient reconstitution of the CD8^+^ T-cell repertoire [6, 7]. Similarly, in immunocompromised mice, the transfer of MCMV-specific CD8^+^ T cells plays a protective role [3, 8, 9]. In contrast to that of T cells, the significance of B cells during acute CMV infection is less clear [10].

B cells play an important role in the development of the architecture of the spleen, particularly in the maintenance of the marginal zone (MZ) [11]. The splenic MZ is a distinctive and specialized structure located at the interface between the lymphoid white pulp and the scavenged red pulp of the spleen. Upon entry into the body, blood-borne viruses such as CMV first interact with immune cells in the splenic MZ. Here, the interplay between innate and adaptive immune mechanisms ensures an early and efficient response to pathogen-associated antigens [12, 13]. The splenic MZ plays an important role in the immune response to blood-borne viral infections because it is enriched with antigen-presenting cells (APCs), including CD169^+^ marginal metallophilic macrophages (MMMs), SIGN-R1^+^ MZ macrophages (MZMs) and conventional dendritic cells (cDCs). Collectively, these cells capture antigens from the circulation and process them for clearance or presentation to cells of the adaptive immune response [14–16]. CD169^+^ macrophages are reported to be the primary targets of viral infection [17–20]. CD169, expressed on the surface of MMMs, is involved in cell‒cell contact and transfers viral antigens to CD8^+^ cDC1s, which in turn prime the antiviral CD8^+^ T-cell response [21]. MMMs also promote viral replication, which can actually increase the delivery of viral antigens to T and B cells and strengthen the antiviral immune response [22, 23]. cDCs are commonly classified into type 1 and type 2 lineages (cDC1s and cDC2s, respectively) [24], which can be further subdivided into several unique cDC subsets with various immune-modulatory roles [25–29]. One of these subsets is Langerin^+^ cDC1s, which are professional cross-presenting subgroups of CD8^+^ cDC1s that express high levels of DEC205, Clec9a, and CD36 and can assimilate circulating apoptotic cell-associated antigens [30]. In addition, upon systemic stimulation, Langerin^+^ cDC1s produce large amounts of IL-12, a cytokine that is important for the clearance of viral and bacterial infections [31, 32]. However, our understanding of whether MMMs and Langerin^+^ cDC1s can modulate primary antiviral CD8^+^ T-cell responses is limited. Moreover, the relationships among B cells, MMMs, and Langerin^+^ cDC1s are unclear.

Here, we demonstrated that B cells are indirectly required for the effective initiation of the antiviral CD8^+^ T-cell response, mainly because the absence of B cells leads to a reduction in Langerin^+^XCR1^+^ cDC1s, which are essential for the priming of a robust MCMV-specific CD8^+^ T-cell response. Moreover, we found that B cells are required to maintain MMMs first by expressing lymphotoxin β (LTβ), which in turn maintains the homeostasis of Langerin^+^XCR1^+^ cDC1s in the splenic MZ. By unbiased ligand‒receptor network analysis, we identified the VCAM1–ITGA4/ITGB1–mediated interaction between MMMs and Langerin⁺ cDC1s. Taken together, these results provide a mechanistic explanation for how B cells regulate the primary MCMV-specific CD8^+^ T-cell response, which may be applicable to other disease models.

## Results

### B cells enhance primary CD8^+^ T-cell responses to MCMV infection

**Fig. 1:**
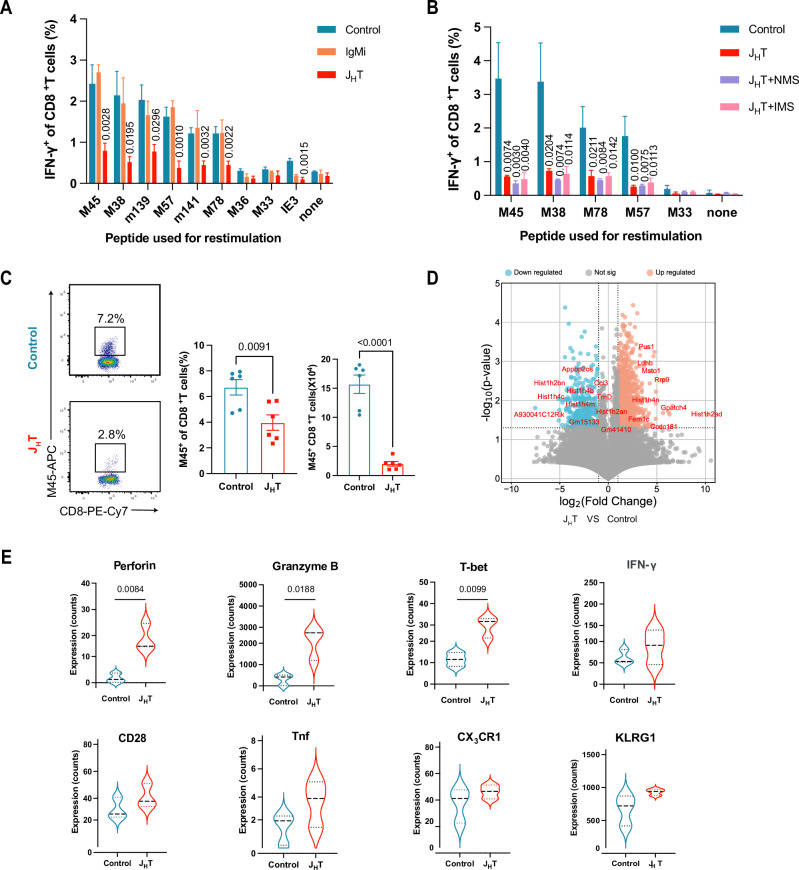
B-cell deficiency attenuates the primary CD8^+^ T-cell response to MCMV infection. **A** Frequency of IFN-γ-producing CD8^+^ T cells in the spleen after stimulation with MCMV peptides at 7 days post-infection in control, J_H_T, or IgMi mice (*n* = 3–12). **B** Frequencies of IFN-γ-producing CD8^+^ T cells in the spleens of MCMV-infected J_H_T and control mice after stimulation with MCMV peptides. Two of the conditions involved treating J_H_T mice with normal mouse serum (NMS) or infected mouse serum (IMS) before MCMV infection (*n* = 3–4). **C** Representative flow cytometry dot plots of MCMV-M45 tetramer^+^CD8^+^ T cells in the spleens of MCMV-infected control and J_H_T mice at 7 days post-infection. The bar graphs show the frequencies (left) and absolute numbers (right) of MCMV-M45 tetramer^+^CD8^+^  T cells in each group (*n* = 6). **D** Volcano plots depicting differentially expressed genes (DEGs) in the splenic MCMV-M45 tetramer^+^CD8^+^ T cells of MCMV-infected J_H_T versus MCMV-infected control mice (P value versus log2-fold change). The top 10 upregulated and downregulated DEGs are labeled. Genes with an adjusted *P* value < 0.05 and log2 (fold change) > 1 or < -1 were considered significantly differentially expressed. **E** Violin plots showing the expression of selected genes associated with the antiviral activity of T cells in splenic MCMV-M45 tetramer^+^CD8^+^ T cells. The data are presented as the means ± SEMs and are representative of two (1A, 1B) independent experiments or pooled from two (1 C) independent experiments. Bulk RNA sequencing data were obtained from three control and three J_H_T mice. Statistical analysis: one-way ANOVA, Fig. 1A, 1B. Student’s *t*-test, Fig. 1C

To explore the role of B cells in the primary CD8^+^ T-cell response, we first infected control and B-cell-deficient J_H_T mice with MCMV. After seven days, we isolated splenocytes from the infected mice and stimulated them with previously reported antigenic MCMV peptides in vitro [33]. The activation of CD8^+^ T cells was assessed by measuring their capacity to produce IFN-γ. Compared with infected control mice, MCMV-infected J_H_T mice presented markedly lower frequencies of IFN-γ-producing CD8^+^ T cells (Fig. 1A). To determine whether the decrease in the primary CD8^+^ T-cell response in J_H_T mice was due to a lack of antibodies, we also infected IgMi mice with MCMV. IgMi mice harbor a polyclonal population of B cells but are not able to class switch or produce secreted antibodies [34]. We found that the percentage of IFN-γ-producing CD8^+^ T cells in the IgMi mice was similar to that in the controls (Fig. 1A). To directly examine the role of immunoglobulins in the acute MCMV-specific CD8^+^ T-cell response, we treated J_H_T mice with normal mouse serum (NMS) or mouse serum from MCMV-infected wild-type (WT) mice (IMS). In agreement with the results of the IgMi infection experiment, the transfer of serum from infected WT mice did not restore the virus-specific CD8^+^ T-cell response during acute MCMV infection (Fig. 1B). Together, these data indicate that secreted antibodies do not play a role in the primary anti-CMV CD8^+^ T-cell response. Next, we assessed whether the reduction in virus-specific activated CD8^+^ T cells affected viral replication. To this end, we isolated the lymph nodes (LNs) of MCMV-infected control and J_H_T mice and quantified viral transcription. Indeed, the weakened virus-specific CD8^+^ T-cell response in the J_H_T mice was accompanied by increased MCMV transcription (Fig. S1A). These findings suggest that early MCMV control is less effective in B-cell-deficient mice than in control mice.

To determine whether the transfer of B cells would restore the magnitude of the primary CD8^+^ T-cell response in B-cell-deficient mice, we reconstituted J_H_T mice with magnetically sorted splenic WT B cells, as previously described [35]. One week after transfer, the mice were infected with MCMV, and the virus-specific CD8^+^ T-cell response was analyzed at 7 days post-infection (Fig. S1B). Notably, adoptive B-cell transfer partially restored the primary CD8^+^ T-cell response of the J_H_T mice (Fig. S1C). To investigate whether the inability of CD8^+^ T cells to respond to MCMV antigens was due to a defect caused by the constitutive lack of B cells from birth, we used our previously published iDTR mice [36] crossed with CD19-Cre [37] and treated them with diphtheria toxin (DT) to ablate B cells. Indeed, inducible depletion of B cells resulted in a dramatically reduced CD8^+^ T-cell response to MCMV antigens upon infection with the virus (Fig. S1, D, E). Together, these findings indicate that the attenuation of the MCMV-specific CD8^+^ T-cell response correlates with the absence of B cells in these mice.

Next, we investigated how a lack of B cells affects virus-specific CD8^+^ T cells in ways other than reducing their IFN-γ production. To this end, we first analyzed CD8^+^ T-cell frequencies in J_H_T mice at steady state and after MCMV infection. We found that the frequency and number of CD8^+^ T cells were comparable in the spleens of J_H_T and control mice at steady state and after MCMV infection (Fig. S1F, G). We subsequently characterized CD8^+^ T cells recognizing the MCMV immunodominant M45 peptide via MHC tetramers in MCMV-infected control and J_H_T mice. Consistent with the reduction in the number of IFN-γ-producing MCMV-specific CD8^+^ T cells, fewer M45 tetramer-positive CD8^+^ T cells were observed in the J_H_T than in the control mice (Fig. 1C). To determine the molecular pathways implicated in the defective CD8^+^ T-cell response during MCMV infection, we sorted equal numbers of these antigen-specific CD8^+^ T cells from each group of mice and subjected them to bulk RNA sequencing. We identified 1,018 genes that were differentially expressed between M45-specific CD8^+^ T cells isolated from J_H_T and control mice (Fig. 1D). Nevertheless, the expression of genes encoding molecules associated with antiviral activity, such as CD28, killer cell lectin-like receptor subfamily G member 1 (KLRG1), perforin, granzyme B, TNF, CX_3_CR1, and IFN-γ, was similar to or even increased in the MCMV-specific CD8^+^ cells of J_H_T mice compared with those of control mice (Fig. 1E). This result indicates that CD8^+^ T cells in J_H_T mice can become fully efficient effector CD8^+^ T cells after recognition of their cognate MCMV antigen. Thus, these results collectively show that the reduction in the magnitude of the primary CD8^+^ T-cell response in J_H_T mice was not due to changes in the effector function of the CD8^+^ T cells themselves. Instead, the increased expression of effector molecules such as perforin and granzyme B by the virus-specific CD8^+^ T cells of the J_H_T mice may be attributed to the increased viral load and the reduced number of virus-specific CD8^+^ T cells. In such a context, existing CD8^+^ T cells may increase their effector functions as a compensatory mechanism.

### Loss of B cells attenuates CD8^+^ T-cell priming after viral infection

**Fig. 2:**
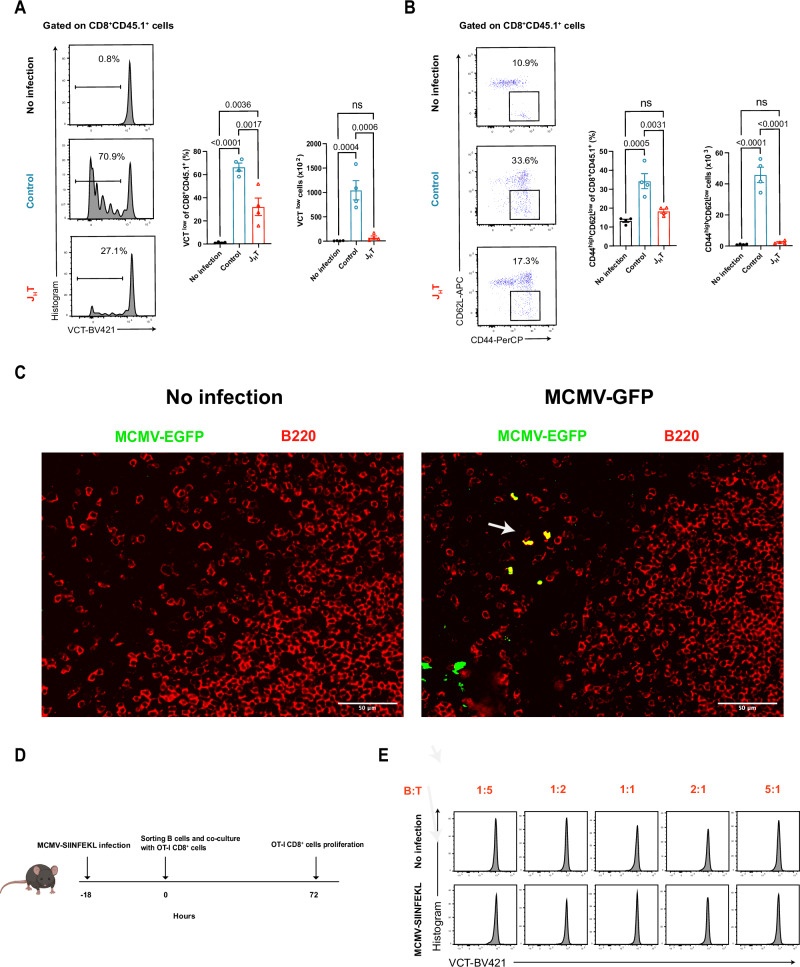
The absence of B cells impairs CD8^+^ T-cell priming after viral infection. **A** Proliferation of adoptively intravenously transferred OVA-specific transgenic OTI CD45.1^+^CD8^+^ T cells at 3 days after the induction of MCMV-SIINFEKL infection. The bar graphs show the frequencies (left) and absolute numbers (right) of proliferating OTI CD45.1^+^CD8^+^ T cells in each group (*n* = 4). **B** Flow cytometry dot plot showing CD62L^-^CD44^+^ effector OTI CD45.1^+^CD8^+^ T cells at 3 days after the induction of MCMV-SIINFEKL infection. The bar graphs indicate the frequencies (left) and absolute numbers (right) of CD62L^-^CD44^+^ effector OTI CD45.1^+^CD8^+^ T cells in each group (*n* = 4). **C** Immunofluorescence images of B220 and MCMV-EGFP in the spleens of WT mice, 48 hours after MCMV-EGFP infection, with uninfected WT mice serving as controls (scale bar, 50 µm; *n* = 4). **D** Schematic diagram outlining the coculture of OVA-specific transgenic OTI CD8^+^ T cells with B cells isolated from MCMV-SIINFEKL-infected WT mice. **E** Flow cytometry histograms showing the proliferation of OVA-specific transgenic OTI CD8^+^ T cells cocultured with B cells isolated from uninfected or MCMV-SIINFEKL-infected WT mice (*n* = 5). The data are presented as the means ± SEMs and are representative of two independent experiments. Statistical analysis: one-way ANOVA, Fig. 2A, 2B

Changes in the function of APCs can impact the virus-specific primary CD8^+^ T-cell response. To assess whether altered APC function was responsible for the reduction in the number of MCMV-specific CD8^+^ T cells in the J_H_T mice, we adoptively transferred Trace-Violet-labeled CD8^+^CD45.1^+^ T cells isolated from OVA-specific TCR-transgenic OT-I mice into CD45.2^+^ J_H_T or control recipients. One day later, the mice were infected with recombinant MCMV encoding an immunodominant OVA peptide (MCMV-SIINFEKL) [38]. At 3 days post-infection, we isolated splenocytes and analyzed the proliferation of OT-I T cells expressing the Vα2Vβ5 OVA-specific TCR (Fig. S2A). Compared with uninfected control animals, infected control animals displayed strong OT-I CD8^+^ T-cell expansion, whereas transferred OT-I CD8^+^ T cells in J_H_T mice proliferated significantly less (Fig. 2A). In addition, the proportion and number of CD62L^−^CD44^+^ effector CD8^+^ T cells were significantly lower among OT-I T cells transferred into J_H_T mice than among those transferred into control mice (Fig. 2B). These results suggest that CD8^+^ T cells cannot be properly primed in J_H_T mice, possibly because of a deficiency of functional APCs in these animals.

B cells express both MHC class I and II molecules and can interact with T cells directly [39, 40]. Thus, B cells can potentially act as APCs during the initiation of the MCMV CD8^+^ T-cell response. To evaluate whether B cells are able to take up antigens after MCMV infection and thus act as direct APCs, we infected WT mice with recombinant MCMV expressing EGFP (MCMV-EGFP) [41]. Forty-eight hours later, splenic histology revealed the colocalization of B cells and MCMV-EGFP signals (Fig. 2C). Flow cytometry data also revealed EGFP expression in B cells after MCMV-EGFP infection, suggesting viral entry and immediate early gene expression (Fig. S2B). To investigate the ability of B cells to prime CD8^+^ T cells, we purified B cells from MCMV-SIINFEKL-infected WT mice and cultured them with OT-I T cells (Fig. 2D). We observed that the B cells isolated from these mice did not induce OT-I CD8^+^ T-cell proliferation, suggesting that they were not the major APC type presenting antigens to CD8^+^ T cells after MCMV infection (Fig. 2E). Together, these data prove that although the defective CD8^+^ T-cell priming observed in the J_H_T mice was due to the lack of B cells, it did not rely on their ability to act as APCs to directly induce MCMV-specific CD8^+^ T-cell responses.

### B-cell deficiency affects the T-cell priming capacity of XCR1^+^CD8^+^ cDC1s

**Fig. 3:**
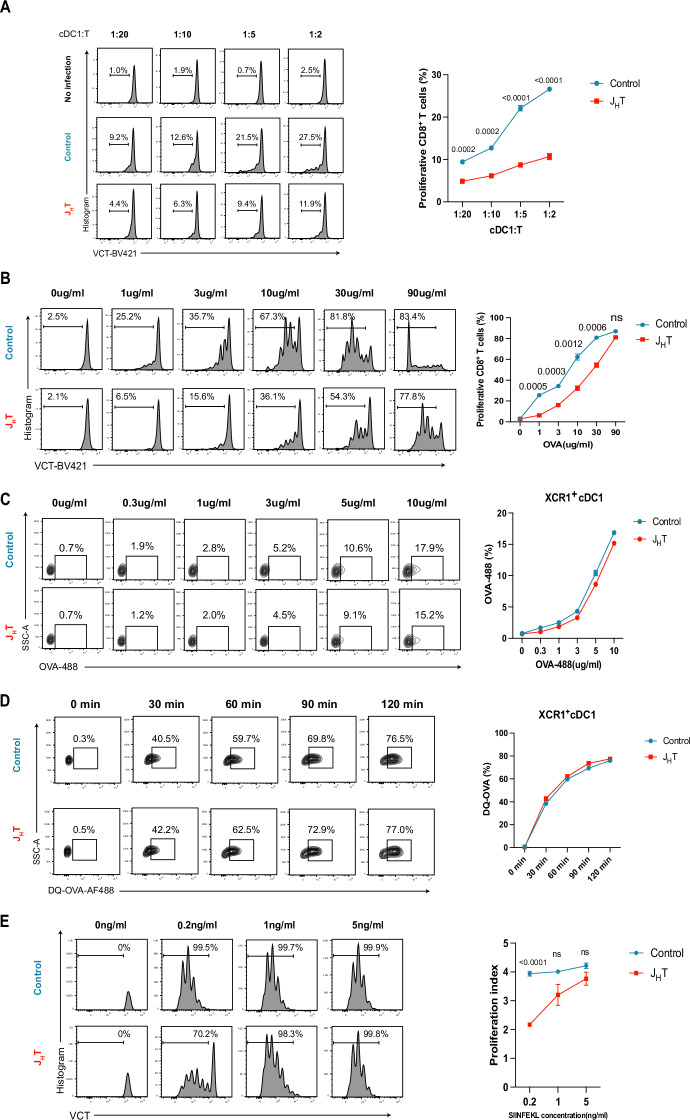
B cells support the CD8^+^ T-cell-priming function of XCR1^+^CD8^+^ cDC1s. **A** Proliferation of OVA-specific transgenic OTI CD8^+^ T cells cocultured at various ratios with cDC1s isolated from uninfected or MCMV-SIINFEKL-infected control and J_H_T mice. The line graph shows the frequencies of proliferating OTI CD8^+^ T cells (*n* = 5). **B** Proliferation of OVA-specific transgenic OTI CD8^+^ T cells cocultured with cDC1s isolated from control or J_H_T mice and pulsed with different concentrations of OVA protein. The line graph indicates the frequencies of proliferating OTI CD8  T cells (*n* = 5). **C** Flow cytometry plots showing the frequencies of OVA-488^+^XCR1^+^ cDC1s at each incubated concentration of OVA-488. Briefly, cDC1s isolated from control or J_H_T mice were incubated with increasing concentrations of Alexa-Fluor-488-labeled OVA for 30 minutes at 37 °C and 5% CO_2_. The line graph shows the frequencies of OVA-488^+^XCR1^+^ cDC1s in each group (*n* = 4). **D** Representative flow cytometry plots of DQ-OVA^+^XCR1^+^ cDC1s exposed to DQ-OVA for different durations. Briefly, cDC1s isolated from control or J_H_T mice were incubated with 10 μg/ml DQ-OVA for 0, 30, 60, 90, or 120 minutes at 37 °C and 5% CO_2_. Line graph indicating the frequencies of DQ-OVA^+^XCR1^+^ cDC1s in each group (*n* = 4). **E** Proliferation of OTI CD8^+^ T cells cocultured with cDC1s isolated from control or J_H_T mice and pulsed with the SIINFEKL peptide. The line graph indicates the proliferation index of OTI CD8^+^ T cells (*n* = 6). Data are presented as the mean ± SEM and are representative of two (3A-3E) independent experiments. Statistical analysis: Student’s *t* test, Fig. 3A–E

Having excluded the possibility that B cells act as APCs to prime MCMV-specific CD8^+^ T cells directly, we presumed that the absence of B cells might affect other APCs central to this process, such as DCs. To this end, we first investigated whether the absence of B cells could change the ability of cDC1s to present MCMV Ags obtained in vivo. We infected control and J_H_T mice with MCMV-SIINFEKL, purified XCR1^+^CD8^+^ cDC1s from the spleen after 18 hours, and cocultured them with CD8^+^ T cells from OT-I mice for 3 days at various ratios (Fig. S3A). We found that coculturing OT-I CD8^+^ T cells with XCR1^+^CD8^+^ cDC1s isolated from infected J_H_T but not control mice significantly reduced their proliferation (Fig. 3A). To further confirm that B-cell deficiency affects the APC function of CD8^+^ cDC1s, we isolated XCR1^+^ cDC1s from the spleens of naïve J_H_T and control mice and incubated them with OVA protein. After 1 h, the XCR1^+^ cDC1s were washed and cocultured with OT-I CD8^+^ T cells. After 3 days, XCR1^+^ cDC1s from J_H_T mice expanded OT-I CD8^+^ T cells significantly less than those from control mice did (Figs. 3B and S3B), demonstrating their inferior antigen-presenting capacity. In addition, the expression of the costimulatory molecules CD40, CD80, and CD86 was significantly lower in the splenic cDC1s of J_H_T mice than in those of control mice, which was consistent with impaired antigen-presenting function (Fig. S3C).

To better understand how the antigen-presenting function of XCR1^+^ cDC1s was altered in J_H_T, we evaluated their uptake of OVA conjugated to Alexa Fluor 488 in vitro. We observed similar levels of fluorescence in the XCR1^+^ cDC1s of J_H_T and control mice, indicating that both exhibited similar antigen uptake patterns (Fig. 3C). To determine whether the XCR1^+^ cDC1s of J_H_T mice presented any antigen processing defects, which may have hampered MHC class I-mediated antigen presentation, we incubated XCR1^+^ cDC1s with OVA conjugated to pH-insensitive BODIPY FL dye (DQ-OVA). After proteolytic degradation, the processed elements of DQ-OVA emit fluorescence, which can be analyzed by flow cytometry. We did not detect any differences in fluorescence between the groups, indicating that the splenic XCR1^+^ cDC1s of J_H_T and control mice were equally capable of processing phagocytosed DQ-OVA (Fig. 3D). Together, these data suggest that the cDC1s of B-cell-deficient mice acquire and process antigens similarly to those of control mice. To examine other possible effects of B-cell deletion on cDC1 function subsequent to antigen phagocytosis and processing, we cocultured XCR1^+^ cDC1s and OT-I CD8^+^ T cells in the presence of varying SIINFEKL peptide concentrations. We found that OT-I CD8^+^ T cells cocultured with XCR1^+^ cDC1s from J_H_T mice expanded less at low SIINFEKL concentrations than those cultured with XCR1^+^ cDC1s from control mice did (Fig. 3E). Accordingly, the number of proliferating OT-I CD8^+^ T cells was significantly lower in cocultures with XCR1^+^ cDC1s from J_H_T mice than in those with control XCR1^+^ cDC1s (Fig. S3D). Taken together, these data indicate that B cells contribute to the ability of splenic XCR1^+^ cDC1s to present antigens to activate CD8^+^ T cells.

### Loss of B cells reduces the number of Langerin^+^XCR1^+^ cDC1 subsets essential for priming the MCMV-specific CD8^+^ T-cell response

**Fig. 4:**
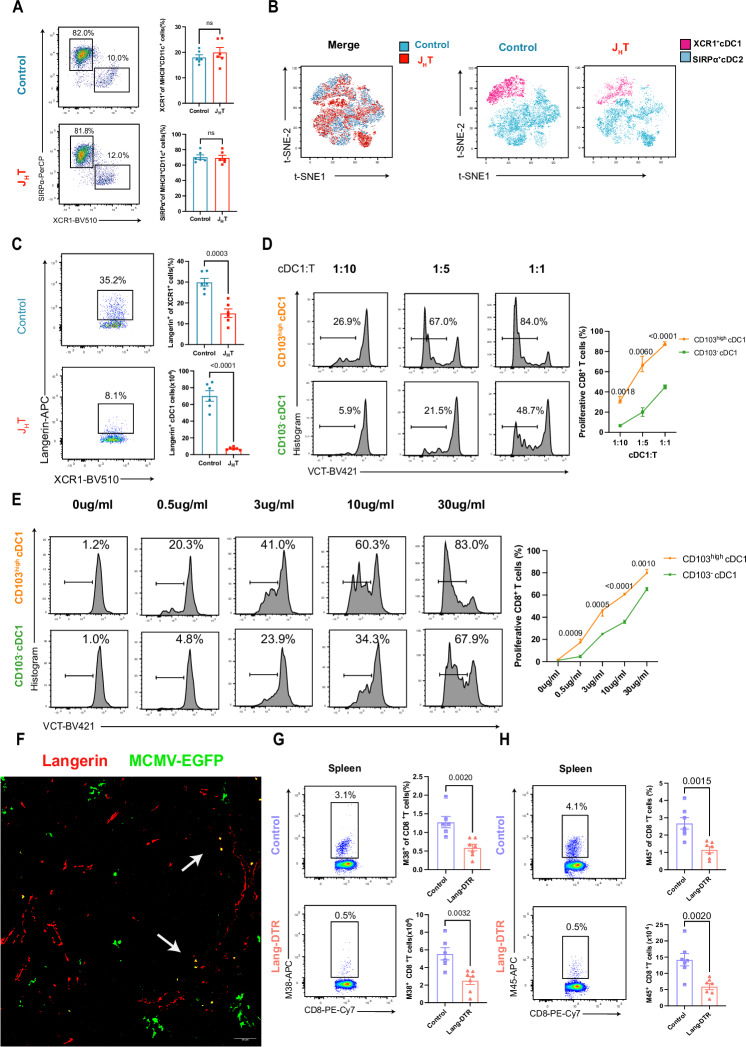
B cells maintain the homeostasis of splenic Langerin^+^ cDC1s. **A** Flow cytometry dot plot of XCR1^+^ cDC1 and SIRPα^+^ cDC2 subsets in the spleens of control or J_H_T mice. The bar graphs indicate the frequencies of XCR1^+^ cDC1s (top) or SIRPα^+^ cDC2s (bottom) (*n* = 6). **B** Three control and three J_H_T concatenated samples, each containing 5000 splenic cDCs (CD11c^+^MHCII^+^CD19^-^), were embedded via t-distributed stochastic neighbor embedding (t-SNE); cDC1s (pink) and cDC2s (blue) were classified according to XCR1 and SIRPα expression (*n* = 3). **C** Flow cytometry dot plot of splenic Langerin^+^ cDC1s from control or J_H_T mice. The bar graphs show the frequencies (top) and absolute numbers (bottom) of Langerin^+^ cDC1s (*n* = 6). **D** Proliferation of OVA-specific transgenic OTI CD8^+^ T cells cocultured at various ratios with CD103^high^ cDC1s or CD103^-^ cDC1s isolated from MCMV-SIINFEKL-infected WT mice. The line graph indicates the frequencies of proliferating OTI CD8^+^ T cells (*n* = 9). **E** Proliferation of OVA-specific transgenic OTI CD8^+^ T cells cocultured with CD103^high^ cDC1s or CD103^-^ cDC1s isolated from WT mice and pulsed with different concentrations of OVA protein. The line graph indicates the frequencies of proliferating OTI CD8^+^ T cells (*n* = 8). **F** Immunofluorescence images of Langerin and MCMV-EGFP in the spleens of WT mice 48 hours after MCMV-EGFP infection (scale bar, 50 µm; *n* = 4). **G** Representative flow cytometry dot plots of splenic MCMV-M38 tetramer^+^CD8^+^ T cells in MCMV-infected control and MCMV-infected Lang-DTR mice. The bar graphs indicate the frequency (top) and absolute number (bottom) of MCMV-M38 tetramer^+^CD8^+^ T cells (*n* = 7). **H** Flow cytometry plot of splenic MCMV-M45 tetramer^+^CD8^+^ T cells in MCMV-infected control and MCMV-infected Lang-DTR mice. The bar graphs show the frequencies (top) and absolute numbers (bottom) of MCMV-M45 tetramer^+^CD8^+^ T cells (*n* = 7). The data are presented as the means ± SEMs and were pooled from two (4A and 4C) independent experiments or representative of two (4B, 4D-4G) independent experiments. Statistical analysis: Student’s *t* test, Fig. 4A, 4C, 4D, 4E, 4G, and 4H

Given that B-cell deficiency reduces the CD8^+^ T-cell priming capacity of cDC1s, we next explored the underlying mechanisms involved. First, we found that the lack of B cells did not affect the frequency of cDCs in the spleen (Fig. S4A). Furthermore, there was no significant difference in the frequencies of the SIRP-α^+^ cDC2 and XCR1^+^ cDC1 subsets in the spleens of the J_H_T and control mice (Fig. 4A). We then employed high-dimensional flow cytometry to characterize cDC subsets in the spleen better, aiming to understand how they might be influenced by B-cell deficiency. We subsequently used t-distributed stochastic neighbor embedding (t-SNE) to show that the composition of the splenic cDC1 and cDC2 populations differed significantly between J_H_T and control mice (Fig. 4B). Further characterization of the cDC1 and cDC2 subsets (Fig. S4B) revealed that the most significantly altered splenic cDC1 and cDC2 subpopulations in the J_H_T mice were Langerin^+^XCR1^+^ cDC1s and endothelial cell-specific adhesion marker (ESAM)^hi^ SIRP-α^+^ cDC2s, respectively. In line with the t-SNE data, flow cytometry analysis of cDC1s and cDC2s revealed that the frequencies and numbers of Langerin^+^XCR1^+^ cDC1s and ESAM^hi^ SIRP-α^+^ cDC2s in the J_H_T mice were significantly lower than those in the control mice (Figs. 4C and S4C). Accordingly, adoptive transfer of B cells into J_H_T mice partially rescued the Langerin^+^XCR1^+^ cDC1 subpopulation (Fig. S4D), as it restored the MCMV-specific CD8^+^ T-cell response.

Next, we asked whether the reduction in Langerin^+^ cDC1s was responsible for the attenuated ability of splenic XCR1^+^CD8^+^ cDC1s to prime T cells in J_H_T mice. In splenic CD8^+^ cDC1s, Langerin expression is mainly intracellular, and interestingly, the expression levels of the costimulatory molecules CD80 and CD86 are slightly higher in the Langerin^+^ cDC1 subset than in the Langerin^-^ cDC1 subset at steady state [30, 42, 43], which may suggest that Langerin^+^ cDC1s are functionally more mature. To assess whether Langerin^+^ cDC1s efficiently present MCMV antigens that they take up in vivo, we infected WT mice with MCMV-SIINFEKL, purified Langerin^+^ cDC1s from the spleen 18 hr later, and cocultured them with OT-I CD8^+^ T cells. Considering that Langerin expression in splenic cDC1s is mainly intracellular and that Langerin^+^ cDC1s coexpress high levels of the integrin CD103 [14, 42], we used CD103 as a sorting marker for the Langerin^+^ cDC1 subset. After 72 h of coculture, we observed that compared with CD103^-^ cDC1s, CD103^high^ cDC1s more efficiently expanded OT-I CD8^+^ T cells (Fig. 4D). To investigate the antigen-presenting function of Langerin^+^ cDC1s in more detail, we sorted CD103^high^ cDC1s and CD103^-^ cDC1s and cultured them with DQ-OVA. We found that the processed elements of DQ-OVA emit fluorescence similarly in CD103^high^ cDC1s and CD103^-^ cDC1s (Fig. S4E), indicating that Langerin^+^ cDC1s and Langerin^-^ cDC1s have comparable abilities to acquire and process antigens. Next, we isolated CD103^high^ cDC1s and CD103^-^ cDC1s from the spleens of WT mice and cocultured them with OT-I CD8^+^ T cells in vitro in the presence of OVA protein. With this experimental setup, we readily detected that CD103^high^ cDC1s were able to induce more proliferation of CD8^+^ T cells than were CD103^-^ cDC1s (Fig. 4E). Together, these results suggest that Langerin^+^ cDC1s exhibit better antigen-presenting ability than Langerin^-^ cDC1s do and that the diminished CD8^+^ T-cell priming function of cDC1s in the spleens of J_H_T mice is due to a reduction in the Langerin^+^ cDC1 subset.

Given the crucial role of Langerin^+^ cDC1s in antigen presentation, we focused on how they contribute to MCMV infection. Spleen histology of WT mice infected with MCMV-EGFP revealed that Langerin colocalized with MCMV-EGFP signals (Fig. 4F). To determine whether splenic Langerin^+^XCR1^+^ cDC1s are required for the control of MCMV infection, we treated Lang-DTR [44] and control mice with DT and subsequently infected them with MCMV. Multiple DT injections were well tolerated [45] and effectively depleted of Langerin^+^XCR1^+^ cDC1s in the spleen (Fig. S4F). At 7 days post-infection, we assessed the virus-specific CD8^+^ T-cell response via MCMV tetramer staining. The frequency and number of MCMV-specific tetramer-positive CD8^+^ T cells were significantly lower in DTR-treated Lang-DTR mice than in control mice (Fig. 4G, H, and S4G). This finding indicates that the reduction in Langerin^+^XCR1^+^ cDC1s was responsible for the decrease in MCMV-specific CD8^+^ T cells observed in J_H_T mice. Collectively, our data suggest that the absence of B cells reduces the number of splenic Langerin^+^XCR1^+^ cDC1s, which may have altered the antigen-presenting function of the remaining cDC1s and ultimately hampers effective CD8^+^ T-cell priming.

### B-cell-expressed LTβ modulates CD169^+^ MMMs to maintain splenic Langerin^+^ cDC1 homeostasis

**Fig. 5:**
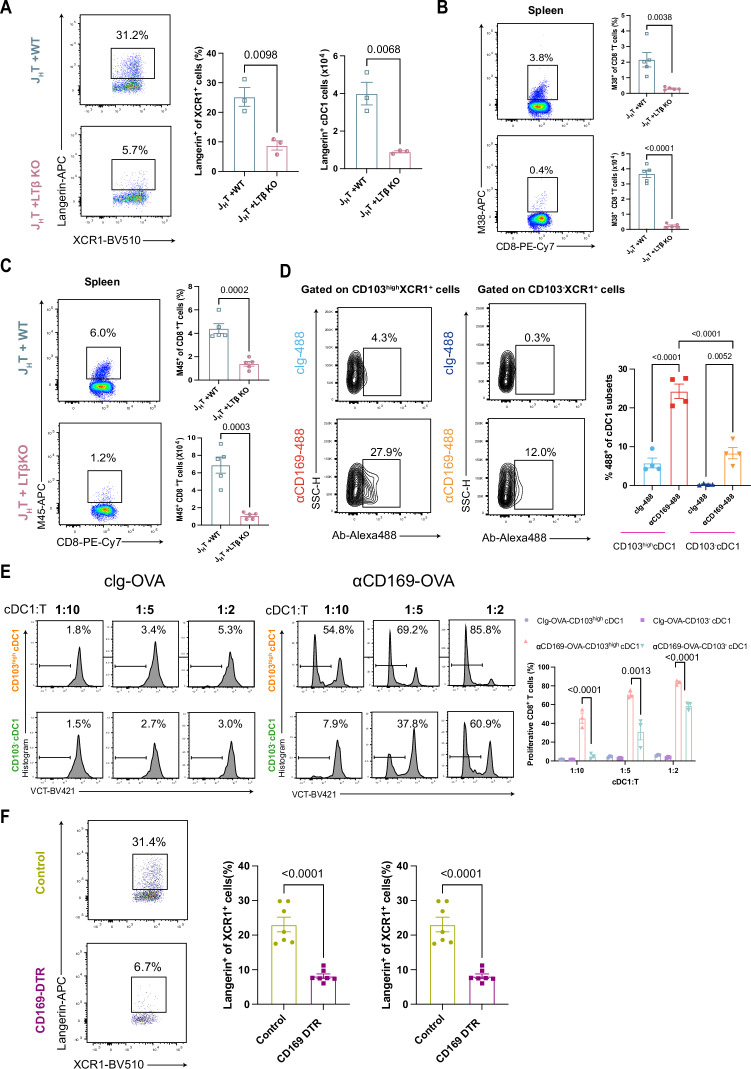
LTβ-expressing B cells maintain MMMs in the spleen to support the homeostasis of splenic Langerin^+^ cDC1s. **A** Flow cytometry plot showing splenic Langerin^+^ cDC1s in J_H_T-mixed WT or J_H_T-mixed LTβ-deficient BM chimeric mice. The bar graphs indicate the frequencies (left) and absolute numbers (right) of Langerin^+^ cDC1s (*n* = 3). **B** Representative flow cytometry dot plots of splenic MCMV-M38 tetramer^+^CD8^+^ T cells in J_H_T-mixed WT or J_H_T-mixed LTβ-deficient BM chimeric mice. The bar graphs show the frequencies (top) and absolute numbers (bottom) of MCMV-M38 tetramer^+^CD8^+^ T cells in each group (*n* = 5). **C** Flow cytometry dot plot showing splenic MCMV-M45 tetramer^+^CD8^+^ T cells in J_H_T-mixed WT or J_H_T-mixed LTβ-deficient BM chimeric mice. The bar graphs show the frequencies (top) and absolute numbers (bottom) of MCMV-M45 tetramer^+^CD8^+^ T cells in each group (*n* = 5). **D** Flow cytometry plot showing Alexa488^+^CD103^high^ cDC1s and Alexa488^+^CD103^-^ cDC1s after the indicated Ab-Alexa488 injection. The bar graphs show the frequencies of Alexa488^+^CD103^high^ cDC1s and Alexa488^+^CD103^-^ cDC1s (*n* = 4). **E** Proliferation of OVA-specific transgenic OTI CD8^+^ T cells cocultured at various ratios with CD103^high^ cDC1s or CD103^-^ cDC1s isolated from the indicated Ab-OVA-immunized WT mice. The bar graphs show the frequencies of proliferating OTI CD8^+^ T cells. **F** Representative flow cytometry dot plots of splenic Langerin^+^ cDC1s from control or CD169-DTR mice. The bar graphs indicate the frequencies (left) and absolute numbers (right) of Langerin^+^ cDC1s (*n* = 7). The data are presented as the means ± SEMs and are representative of two to three independent experiments or pooled from two (5F) independent experiments. Statistical analysis: one-way ANOVA, Fig. 5D and 5E. Student’s *t*-test, Fig. 5A–C, F

We next sought to investigate the mechanisms by which B cells sustain the homeostasis of splenic Langerin^+^ cDC1s. Langerin^+^ cDC1s are predominantly localized in the MZ of the spleen [14, 43], and it has been reported that B cells are able to support the development and maintenance of secondary lymphoid tissues such as the splenic MZ by expressing LTβ [46, 47]. We therefore asked whether B cells could sustain the homeostasis of Langerin^+^ cDC1s by expressing LTβ. To test this hypothesis, we generated mixed BM chimeras from the BM of WT mice mixed with that of J_H_T mice (control) or from the BM of LTβ-deficient mice mixed with that of J_H_T mice, generating mice in which only the B cells were unable to undergo LTβ. As expected, the absence of B-cell-expressed LTβ markedly reduced the Langerin^+^ cDC1 population (Fig. 5A). Next, we infected chimeric mice with MCMV and observed significantly fewer MCMV-tetramer-positive CD8^+^ T cells in the absence of B cells expressing LTβ, mimicking the situation in B-cell-deficient J_H_T mice (Fig. 5B, C). Collectively, these data prove that the absence of B-cell-expressed LTβ impairs CD8^+^ T-cell priming in response to MCMV infection, likely due to an inadequate number of Langerin^+^ cDC1s.

Previous reports have shown that lymphotoxin (LT-α1β2)-LT-βR signaling regulates the development and/or homeostasis of splenic CD11b^+^ DCs but not cDC1s [48–51] and that the absence of B-cell-expressed LTβ also leads to alterations in the homeostasis of other cells in the MZ [47]; therefore, we investigated whether the regulation of Langerin^+^ cDC1s by B-cell-expressed LTβ needs to be dependent on other cells. As previously reported [47, 52, 53], we confirmed that LTβ expressed by B cells is required for the maintenance of MMMs within the splenic MZ (Fig. S5A). Interestingly, when we analyzed the spleens of WT mice at steady state, we found that Langerin^+^ cDC1s colocalized with MMMs (Fig. S5B), and this spatial proximity suggests the possibility of interaction between MMMs and Langerin^+^ cDC1s. Previous studies reported that the functions of MMMs are Ag uptake and depend on CD169 binding to sialic acid on CD8^+^ cDC1s to transfer Ag to CD8^+^ cDC1s for CD8^+^ T-cell priming [21, 54]. Given that Langerin^+^ cDC1s are a subset of CD8^+^ cDC1s, we hypothesized that MMMs can transfer antigens directly to them. To test this hypothesis, we injected WT mice with an Alexa 488-labeled anti-CD169 (αCD169- AF488) antibody along with anti-CD40 and PolyI:C as adjuvants and analyzed the transfer of αCD169- AF488 from MMMs to Langerin^+^ cDC1s via fluorescence microscopy and flow cytometry. Indeed, we confirmed that MMMs are able to transfer antigens to Langerin^+^ cDC1s (Fig. S5C). Moreover, we found that MMMs transferred more antigens to the CD103^high^ cDC1 subset than to the CD103^-^ cDC1 subset (Fig. 5D). Finally, we sorted CD103^high^ and CD103^-^ cDC1s from WT mice injected with anti-CD169-OVA (αCD169-OVA) and used them to stimulate OT-I CD8^+^ T cells in vitro. In line with our previous results, OT-I CD8^+^ T cells cocultured with CD103^high^ cDC1s presented significantly increased proliferation compared with their CD103-negative counterparts (Fig. 5E), further supporting the concept that the Langerin^+^ cDC1 subset is able to acquire more antigens from MMMs and present antigens more efficiently to CD8^+^ T cells.

Having established an intimate interaction between Langerin^+^ cDC1s and MMMs, our next objective was to determine whether B-cell-expressed LTβ-regulated Langerin^+^XCR1^+^ cDC1 homeostasis is dependent on MMMs. Cytofluorometric analysis of DT-treated CD169-DTR mice revealed that MMM depletion significantly decreased the frequency and number of Langerin^+^XCR1^+^ cDC1s (Fig. 5F) but did not alter the presence of B cells (Fig. S5, D, E), indicating that MMMs play an essential role in maintaining splenic Langerin^+^XCR1^+^ cDC1 homeostasis and that the regulation of Langerin^+^XCR1^+^ cDC1 homeostasis by B cells is dependent on MMMs. Moreover, in DT-treated Lang-DTR mice, Langerin^+^ cDC1 depletion did not affect the population of MMMs (Fig. S5F). Collectively, these data suggest that the lack of B-cell-expressed LTβ disrupts the architecture of the spleen, particularly the localization of MMMs in the MZ, which in turn leads to a reduction in Langerin^+^XCR1^+^ cDC1s. Notably, we cannot exclude the possibility that B cells also have the potential to regulate the homeostasis of Langerin^+^ cDC1s directly through the expression of LTβ, but this study is difficult to investigate because the inability of B cells to express LTβ leads to a significant reduction in MMMs, which in turn disrupts Langerin^+^ cDC1 homeostasis.

As in B-cell-deficient mice, the antigen processing (Fig. S5G) abilities of cDC1s were not affected by the depletion of MMMs. Nevertheless, when XCR1^+^ cDC1s from the spleens of naïve DT-treated CD169-DTR or control mice were loaded with OVA protein and then cocultured with OT-I CD8^+^ T cells, as in the J_H_T mouse experiments, MMM depletion reduced the ability of XCR1^+^ cDC1s to prime OVA-specific OT-I CD8^+^ T cells (Fig. S5H). Taken together, these data suggest that depletion of CD169^+^ MMMs perturbs the homeostasis of Langerin^+^XCR1^+^ cDC1s and affects the ability of XCR1^+^ cDC1s to prime CD8^+^ T cells. These data clearly demonstrate that the ability of B cells to maintain splenic Langerin^+^ cDC1 homeostasis is dependent on the self-expression of LTβ to sustain MMMs.

### VCAM1–ITGA4/ITGB1 interaction coordinates MMMs–Langerin^+^XCR1^+^ cDC1 cross-talk

**Fig. 6:**
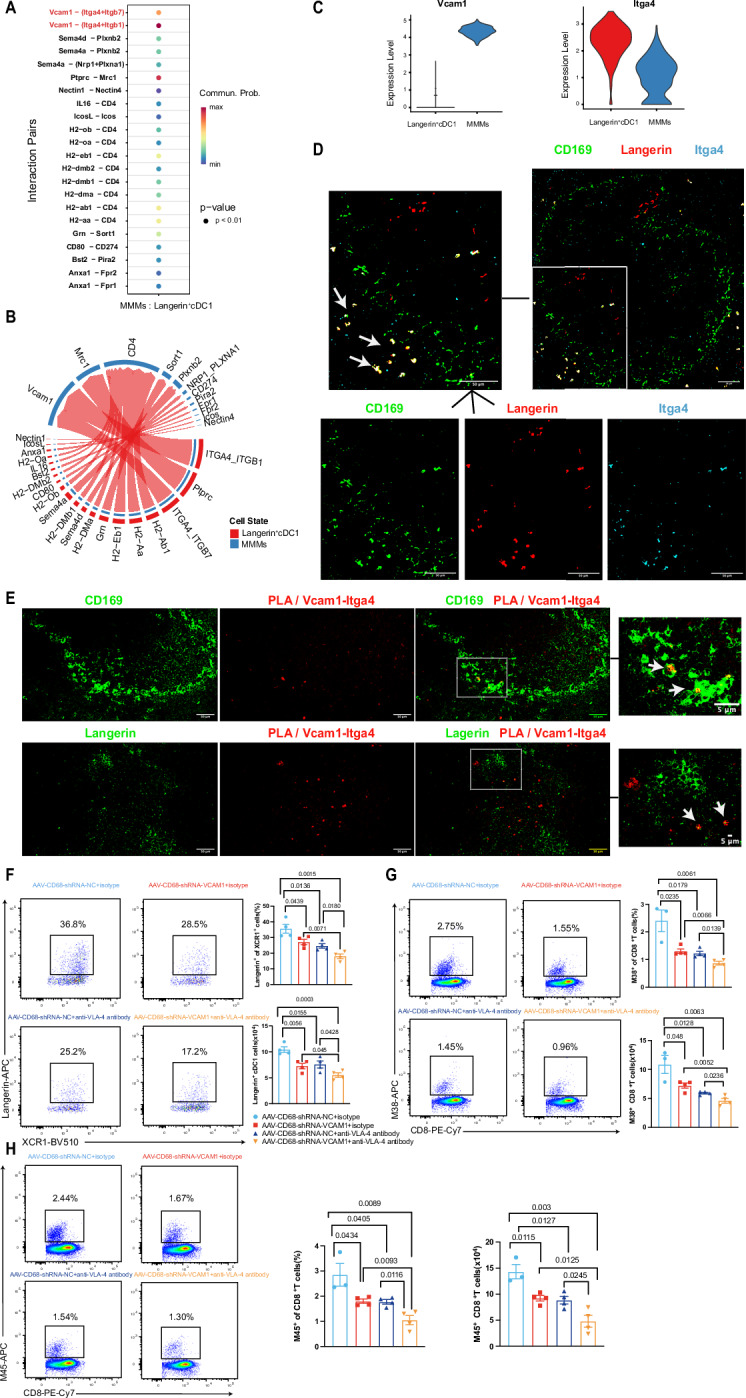
MMMs cross-talk with Langerin^+^XCR1^+^ cDC1s via VCAM1-ITGA4/ITGB1. **A** Ligand‒receptor pair analysis between MMMs and Langerin^+^XCR1^+^ cDC1 clusters. **B** Chord diagram showing preferential interactions between MMMs and Langerin^+^XCR1^+^ cDC1 clusters. **C** Violin plots of VCAM1 and ITGA4 expression in the indicated cell subsets. **D** Immunofluorescence images of CD169 (Siglec-1), Langerin, and ITGA4 in the spleens of WT mice (scale bar, 50 µm; *n* = 4). **E** The interaction of VCAM1 and ITGA4 was visualized by PLA with additional staining for CD169 (Siglec-1) and Langerin (scale bar, 50 µm or 5 µm; *n* = 4). **F** Representative flow cytometry dot plots of splenic Langerin^+^ cDC1 cells from AAV-CD68-shRNA-NC+isotype (upper left), AAV-CD68-shRNA-VCAM1+isotype (upper right), AAV-CD68-shRNA-NC+anti-VLA-4 antibody (bottom left), and AAV-CD68-shRNA-VCAM1+anti-VLA-4 antibody (bottom right)-injected mice. The bar graphs show the frequencies (top) and absolute numbers (bottom) of Langerin^+^ cDC1s (*n* = 4). **G** Representative flow cytometry dot plots of splenic MCMV-M38 tetramer^+^ CD8^+^ T cells in the AAV-CD68-shRNA-NC+isotype (upper left), AAV-CD68-shRNA-VCAM1+isotype (upper right), AAV-CD68-shRNA-NC+anti-VLA-4 antibody (bottom left), and AAV-CD68-shRNA-VCAM1+anti-VLA-4 antibody (bottom right)-injected mice. The bar graphs show the frequencies (top) and absolute numbers (bottom) of MCMV-M38 tetramer^+^CD8^+^ T cells in each group (*n* = 3-4). **H** Flow cytometry dot plot showing splenic MCMV-M45 tetramer^+^CD8^+^ T cells in the AAV-CD68-shRNA-NC+isotype (upper left), AAV-CD68-shRNA-VCAM1+isotype (upper right), AAV-CD68-shRNA-NC+anti-VLA-4 antibody (bottom left), and AAV-CD68-shRNA-VCAM1+anti-VLA-4 antibody (bottom right)-injected mice. The bar graphs show the frequencies (left) and absolute numbers (right) of MCMV-M45 tetramer^+^CD8^+^ T cells in each group (*n* = 3, 4). The data are presented as the means ± SEMs. Statistical analysis: one-way ANOVA, Fig. 6F–H

Next, we investigated how MMMs maintain Langerin^+^ cDC1 homeostasis in the spleen. For this purpose, we sorted CD11c^+^ spleen cells from WT mice and performed single-cell sequencing (scRNA-Seq). On the basis of previously defined splenic CD11c^+^ cell signatures and markers, we defined cDC1s, cDC2s, plasmacytoid DCs (pDCs), monocytes, and macrophages in our collected dataset (Fig. S6A). We subsequently selected MMMs and Langerin^+^ cDC1s (Fig. S6B) and inferred the presence of cross-talk between MMMs and Langerin^+^ cDC1s on the basis of their ligand‒receptor interactions from the analysis of the scRNA-seq data. Individual ligand‒receptor pair analysis suggested that VCAM1-ITGA4/ITGB1 was the top-ranking ligand pair between MMMs and Langerin^+^ cDC1s in WT mice (Fig. 6A, B). Accordingly, VCAM1 and ITGA4 were highly expressed in MMMs and Langerin^+^ cDC1 clusters, respectively (Figs. 6C and S6C). To explore whether VCAM1–ITGA4/ITGB1 interactions contribute to the cross-talk between MMMs and Langerin⁺XCR1⁺ cDC1s, we first examined their spatial organization in the spleen. We observed that CD169, Langerin, and ITGA4 colocalized in the spleens of WT mice, as determined by fluorescence microscopy (Fig. 6D). To further assess potential molecular interactions, we performed an in situ proximity ligation assay (PLA). PLA revealed specific VCAM1–ITGA4 interaction signals (Fig. 6E), and more importantly, the PLA signals colocalized with CD169⁺ MMMs and Langerin⁺ cDC1s (Fig. 6E), suggesting that the VCAM1–ITGA4/ITGB1 adhesion axis may mediate physical contact between these two cell types. To further understand whether MMMs can regulate the homeostasis of Langerin^+^ cDC1s via the VCAM1-ITGA4/ITGB1 interaction, we treated WT mice with a blocking anti-VCAM1 antibody. While we did not observe any changes in the percentage or number of MMMs after anti-VCAM1 antibody treatment (Fig. S7A), VCAM1 expression on MMMs was significantly reduced (Fig. S7B). Notably, our data indicate that the frequencies and numbers of splenic Langerin^+^XCR1^+^ cDC1s were significantly decreased after VCAM1 blockade (Fig. S7C). Next, we infected anti-VCAM1 antibody-treated mice with MCMV and assessed the virus-specific CD8^+^ T-cell response at 7 days post-infection. We found that the frequency and number of MCMV-specific tetramer-positive CD8^+^ T cells were significantly lower in the anti-VCAM1 antibody-treated mice than in the isotype control-treated mice (Fig. S7, D, E).

Previous studies have indicated that in addition to MMMs, splenic endothelial cells also exhibit high levels of VCAM1 expression [55, 56]. To eliminate VCAM1 expression specifically in macrophages, we used AAV-CD68-shRNA-VCAM1. Furthermore, to determine whether blockade of the VCAM1 receptor VLA-4 recapitulates the effects of VCAM1 blockade and whether simultaneous interference with both molecules produces additive or synergistic outcomes, we administered VLA-4–blocking antibodies to mice receiving either AAV-CD68-shRNA-NC or AAV-CD68-shRNA-VCAM1. Using flow cytometry, we confirmed that, compared with the control, the injection of AAV-CD68-shRNA-VCAM1 reduced the expression of VCAM1 in MMMs (Fig. S7F). Correspondingly, VLA-4 blockade induced robust compensatory upregulation of VCAM1 in MMMs (Fig. S7F). Consistent with anti-VCAM1 antibody treatment, knocking down VCAM1 in MMMs did not affect the proportion or number of MMMs (Fig. S7G). Next, we assessed the consequences of perturbing VCAM1 or VLA-4 on the splenic Langerin^+^ cDC1 compartment. Both knocking down VCAM1 in MMMs and VLA-4 blockade individually resulted in a marked reduction in the frequency and absolute number of Langerin⁺ cDC1s, whereas concurrent disruption of both pathways yielded an even more pronounced loss of this subset (Fig. 6F). Following MCMV infection, the frequency of MCMV-specific tetramer⁺CD8⁺ T cells was reduced in mice in which VCAM1 was downregulated in the MMMs or VLA-4 blockade alone, with the decrease further increasing under combined treatment (Fig. 6G, H, S7, H, I). Taken together, these findings provide evidence that VCAM1–ITGA4/ITGB1 interactions facilitate the formation of a cellular interface between MMMs and Langerin⁺XCR1⁺ cDC1s, thereby supporting their coordinated cross-talk within the splenic microenvironment and promoting effective antiviral CD8⁺ T-cell responses.

## Discussion

CMV infection is an important cause of increased morbidity and mortality among immunocompromised individuals, such as recipients of hematopoietic cell transplants and solid organ transplants; thus, CMV infection remains a compelling but underaddressed medical problem [7, 57]. Our data reveal how B cells mechanistically contribute to the primary MCMV-specific CD8^+^ T-cell response. In particular, we demonstrated that 1) B cells play an important role in maintaining the homeostasis of Langerin^+^XCR1^+^ cDC1s, which are critical for efficient initiation of the antiviral CD8^+^ T-cell response; 2) the regulation of Langerin^+^XCR1^+^ cDC1 homeostasis by B cells depends on their own expression of LTβ to maintain MMMs first, and MMMs in turn regulate the homeostasis of Langerin^+^XCR1^+^ cDC1s through VCAM1–ITGA4/ITGB1 cross-talk.

In addition to producing antibodies, B cells modulate immune responses in a variety of ways, for example, by producing proinflammatory cytokines such as TNFα and IL-6 or anti-inflammatory cytokines, including IL-10, IL-35, and TGF-β [58–61]. These B-cell-derived cytokines modulate the onset and development of autoimmunity in both mice and humans [62–64]. Moreover, Graalmann et al. reported that B cells modulate the CD8^+^ T-cell response to influenza or modified vaccinia Ankara (MVA) infection [65]. Consistent with these reports, we demonstrated that the absence of B cells from birth reduces the magnitude of the primary CD8^+^ T-cell response to MCMV infection, which can be overcome by the adoptive transfer of B cells. Our data also indicate that the decreased MCMV CD8^+^ T-cell response is not due to the antibody-secreting function of B cells. Many studies have reported that B cells can also serve as professional APCs for CD4^+^ T-cell priming [66–68]; however, although B cells can capture virus-like particles similarly to DCs, they cannot efficiently process exogenous virus-like particles for cross-presentation in association with MHC class I [69]. In the present study, we also observed that B cells do not effectively present antigens to naïve CD8^+^ T cells following MCMV infection, strongly suggesting that B cells modulate the MCMV-specific CD8^+^ T-cell response via mechanisms other than direct antigen presentation.

B cells play crucial roles in the formation and maintenance of the splenic architecture, especially in the MZ. The splenic MZ is enriched in APCs, including MMMs and Langerin^+^ cDC1s. The Langerin^+^ subset among cDC1s is particularly important for cDC1 functions such as cross-presentation and CD4^+^ Th1 cell stimulation [14, 30, 42]. Our studies indicated that the abundance of Langerin^+^ cDC1s was significantly reduced in B-cell-deficient mice. Prendergast et al. revealed that Langerin^+^ cDC1s play a pivotal role in the initiation of the CD8^+^ T-cell response against bacteremia [31]. Here, we used Lang-DTR mice to ablate Langerin^+^ cDC1s, and in accordance with previous reports, we demonstrated that Langerin^+^ cDC1s play a critical role in the primary MCMV-specific CD8^+^ T-cell response. Several reports suggest that MMMs can interact with cDC1s to promote antiviral T-cell responses [21, 70, 71]. By immunizing mice with an αCD169-488 antibody, we confirmed that MMMs can transfer antigens to Langerin^+^ cDC1s; more importantly, our data suggest that MMMs are able to transfer antigens to CD103^high^ (Langerin^+^) cDC1s more efficiently than to CD103^-^ (Langerin^-^) cDC1s. Interestingly, a recent study by Mauvais et al. revealed that MMMs process internalized antigens through an efficient vacuolar pathway and contribute to antitumor immunity independently of cDC1s [72]. These studies strongly support that MMMs play an important role in antiviral CD8^+^ T-cell responses, and indeed, when we depleted MMMs by treating CD169-DTR mice with DT, we found that MCMV-specific CD8^+^ T-cell responses were suppressed (data not shown). By characterizing the Langerin^+^ cDC1s of MMM-depleted mice, we further revealed that the presence of MMMs is essential for maintaining Langerin^+^ cDC1 homeostasis. Thus, MMMs represent a direct link between B cells and Langerin^+^ cDC1s, whereby B-cell-expressed LTβ is a key factor in maintaining MMMs within the MZ [47, 73, 74], which is subsequently critical for the regulation of Langerin^+^ cDC1 homeostasis. Through unbiased ligand‒receptor network analysis, we demonstrated that MMMs shape Langerin^+^ cDC1 homeostasis through the VCAM1-ITGA4/ITGB1 axis, but other interactions between MMMs and Langerin^+^ cDC1s remain to be identified. A previous report showed that the loss of B-cell-expressed LTβ also altered the number of marginal zone stromal cells [47], which are essential for MMM development [75]. Our study further revealed that MMMs play an important role in regulating the homeostasis of Langerin^+^ cDC1s. Taken together, our findings, together with those of previous studies, provide a more detailed picture of how B cells regulate the homeostasis of cells in the MZ. Despite these advances, the downstream signaling mechanisms by which LTβ mediates these effects remain incompletely understood. Canonically, the engagement of LTβR by LTα1β2 activates the noncanonical NF-κB pathway through the stabilization of NF-κB–inducing kinase (NIK). Consistent with this model, myeloid-specific deletion of NIK resulted in a marked reduction in both MMMs and Langerin⁺ cDC1s, providing in vivo genetic evidence for the involvement of LTβ–NIK signaling in maintaining the homeostasis of these cell populations (data not shown). However, a comprehensive dissection of the downstream LTβ signaling cascade will require substantial and dedicated efforts in the future.

Consistent with previous reports, our unsupervised visualization of high-dimensional flow cytometry data revealed that the frequency of ESAM^+^ cDC2s was lower in B-cell-deficient mice than in control mice [50, 76]. Moreover, adoptive transfer of B cells into J_H_T mice partially rescued the ESAM^+^ cDC2 subpopulation (data not shown). cDC2s induce the activation of CD4^+^ T cells and their differentiation into Th2, Th17, and T-follicular helper cell subsets [77–80]. Moreover, ESAM^+^ cDC2s, which constitute the major cDC2 subpopulation in the splenic MZ, are implicated in germinal center formation [48, 81]. Therefore, the use of different disease models to explore whether B cells can modulate CD4^+^ T-cell responses through ESAM^+^ cDC2s represents an intriguing avenue for future research, which is currently hampered by the lack of cDC2-specific cell ablation mouse models.

NK cells are well established as critical mediators of early control during MCMV infection. In C57BL/6 mice, Ly49H⁺ NK cells recognize the viral m157 ligand and expand robustly, thereby limiting viral replication [82, 83]. More importantly, the benefits of the NK cell response to the host extend beyond its direct antiviral effects to include the promotion of adaptive immunity [84]. We found that following MCMV infection, B-cell–deficient mice presented a greater frequency of Ly49H⁺ NK cells, but their absolute numbers remained unchanged. Moreover, the capacity of Ly49H⁺ NK cells to produce IFN-γ upon stimulation was comparable to that of control cells (data not shown). These data indicate that B-cell deficiency likely does not lead to impaired NK cell responses. Importantly, the reduced virus-specific CD8⁺ T-cell immunity in B-cell–deficient mice therefore cannot be explained by alterations in NK cell function but rather highlights the distinctiveness of our investigation into how B cells regulate antigen-presenting cell homeostasis and shape the antiviral CD8⁺ T-cell response.

B-cell-depleting therapies, such as anti-CD20 antibodies, are widely used in patients with autoimmune diseases such as multiple sclerosis [85] and systemic lupus erythematosus [86]. However, such treatments have been associated with an increased risk of viral infections [87], including reactivation of latent cytomegalovirus (CMV) [88], and impair viral vaccine-induced CD8⁺ T-cell expansion and activity [65]. Using a murine model, our study demonstrated that B cells play a crucial role in maintaining the homeostasis of splenic marginal zone antigen-presenting cells, particularly MMMs and Langerin^+^ cDC1s, which are essential for initiating robust antiviral CD8⁺ T-cell responses. These insights may help explain how B-cell depletion impairs antiviral immunity in clinical settings and suggest that restoring antigen-presenting cell function could be a strategy to mitigate the risk of viral infections in patients receiving B-cell-targeted immunotherapies. More interestingly, as mentioned earlier, B-cell depletion is one of the most effective treatments for multiple sclerosis (MS), but the reason for the effectiveness of this therapy is not clear. Furthermore, some studies have shown that CD169^+^ macrophages are abundant in MS patients and promote neuroinflammation, suggesting a key role for CD169^+^ macrophages in the pathophysiology of MS [19, 89]. On the basis of our current findings and the fact that one can find clonal expansion of CD8^+^ T cells in the brains of MS patients [90], we suggest that the amelioration of disease observed in MS patients after B-cell depletion may be due to reduced activation of autoreactive CD8^+^ T cells.

In conclusion, our findings shed new light on the role of B cells in antiviral immunity. In addition to contributing to viral clearance through the production of antibodies and cytokines, our findings suggest that B cells critically contribute to the initiation of the virus-specific CD8^+^ T-cell response by regulating other APCs, such as macrophages and cDCs. These mechanistic insights will broaden our understanding of the multifaceted roles of B cells in shaping adaptive immune responses and offer new strategies for the treatment of immune-mediated diseases.

## Materials and Methods

### Mice

All the mice used were on the C57BL/6J background. The following mice have been previously described: J_H_T [91], IgMi [34], OT-I [92], C57BL/6J [93], and CD45.1 [94], and they were bred in our facility (Translational Animal Research Center of the University Medical Center Mainz) under specific pathogen-free (SPF) conditions. For the OT-I adoptive transfer experiments, we crossed OT-I mice with CD45.1^+^ mice to generate CD45.1^+^OT-I mice. CD169-DTR mice were provided by Lang KS. Lang DTR mice were obtained from Probst HC, and LTβ BM cells were provided by Gommerman JL. For all the experiments, adult mice of both sexes were used, and the appropriate littermate mice were used as WT controls. All animal experiments were performed in accordance with the guidelines of the Central Animal Facility institution.

### MCMV infection

Mice were infected via the left hind footpad with 1×10^5^ plaque-forming units (PFU) of cell culture-derived MCMV (strain Smith) or intravenously (i.v.) injected with 2×10^5^ PFU of MCMV-SIINFEKL [38] or i.v. injected with 1×10^6^ PFU of MCMV-EGFP[41]. Viral gene expression in the draining lymph node was quantified by RT‒qPCR specific for m123/IE1 as described previously [95].

### Preparation of single-cell suspensions and cell sorting

The cells used for analysis of the DC population were purified from the spleen. The spleen was cut into grain-sized pieces and shaken for 30 min at 37 °C in 1 ml of Roswell Park Memorial Institute (RPMI) 1640 media supplemented with 200 U/ml collagenase type IV (Worthington Biochemical) and 0.5 U/ml DNaseI (Roche). After being digested, a cell suspension was obtained by passing through a 70 μm cell strainer and washing in PBS with 10 mM EDTA and 2% fetal calf serum (FCS), followed by centrifugation and lysis with ACK lysis buffer. To isolate MMMs, the spleen was cut into small pieces in polypropylene tubes with 0.75 ml of RPMI supplemented with 4 mg/ml lidocaine (Sigma). In addition, 0.25 ml of RPMI containing 4 WU/ml of Liberase TL (Roche) and 2 U/ml of DNaseI (Roche) was added. The samples were then incubated at 37 °C with constant stirring for 15 minutes until digestion. After being digested, the cells were passed through a 70 μm cell strainer and washed once with ice-cold RPMI plus 10% FCS, 20 mM HEPES, 10 mM EDTA, and 50 μM 2-mercaptoethanol, followed by centrifugation and lysis with ACK lysis buffer. To purify the DCs, we used anti-CD11c magnetic-activated cell sorting (MACS) microbeads (Miltenyi Biotec) according to the manufacturer’s protocol. To sort XCR1^+^CD8^+^ cDC1 populations, purified CD11c^+^ cells were stained for CD8a, CD11c, MHCII, XCR1, SIRPα, and viability dye. The ARIA III cell sorter (BD Biosciences) was subsequently used to sort cells on the basis of the strong expression of CD11c and MHCII, as well as the presence of XCR1 and CD8a. After MACS or ARIA III sorting, the cells were reconfirmed to have >95% purity and viability.

### Flow Cytometry

Single cells were suspended in fluorescence-activated cell sorting (FACS) buffer (PBS/2 mM EDTA/2% FCS) and blocked with anti-mouse CD16/32 (BioLegend) for 15 min at 4 °C. After blocking, the cells were incubated with the appropriate surface antibodies at 4 °C in the dark for 20 minutes. MCMV-specific CD8^+^ T cells were stained with M38 or M45 tetramers at 4 °C in the dark for 40 min. Intracellular staining requires staining at 4 °C for 1 hour or overnight after fixation and permeabilization via a FoxP3 staining kit (Invitrogen) according to the manufacturer’s instructions. We used the following antibodies for flow cytometry: anti-B220 (RA3-6B2), anti-CD4 (GK1.5), anti-CD8a (53-6.7), anti-CD11b (M1/70), anti-CD11c (N419), anti-CD19 (6D5), anti-CD21 (7E9), anti-CD40 (3/23), anti-CD44 (IM7), anti-CD45 (30-F11), anti-CD45.1 (A20), anti-CD62L (MEL-14), anti-CD80 (16-10A1), anti-CD103 (2E7), anti-CD106/VCAM1 (429 (MVCAM. A)), anti-CD172/SIRP alpha (P84), anti-ESAM (1G8/ESAM), anti-F4/80 (BM8), anti-FceRIa (MAR-1), anti-IFN-γ (XMG1.2), anti-Ly-6C (HK1.4), anti-MHCII (M5/114.15.2), anti-TCR V alpha 2 (B20.1), anti-TCR Vbeta5.1 5.2 (MR9-4), anti-XCR1 (ZET) (all from Biolegend), and anti-CD64 (X54-5/7.1),anti-CD86 (GL1), anti-CD135 (A2F10.1), anti-Ly-6G (1A8), anti-IgD (11-26c), anti-IgM (II/41) (all from BD Biosciences), and anti-CD169 (SER-4) (Thermo Fisher Scientific), and anti-CD207/Langerin (122D5.03) (Eurobio Scientific) antibodies. Flow cytometry data were acquired via a FACSCanto II (BD Biosciences) or FACS Symphony (BD Biosciences) and analyzed via FlowJo v10 software.

### Immunofluorescence

Whole spleens were cut into 6–8 mm sections with a cryostat. The sections were dried overnight at room temperature and then stored at −80 °C until staining began. Nonspecific staining was blocked with PBS plus 5% normal goat serum and 0.5% Triton-100 for 1 hour at room temperature, after which the sections were stained at 4 °C overnight with the following primary antibodies: rat anti-CD169 (MOMA-1) (BMA BIOMEDICALS), rat anti-B220 (RA3-6B2) (BD Biosciences), rat anti-CD207/Langerin (eBioL31), and chicken anti-GFP (Novus). The next day, the sections were washed and further labeled with the appropriate secondary antibodies (anti-rat IgG CF555 (Sigma Aldrich) or anti-chicken CF488A (Sigma Aldrich)) for 1 h at RT, followed by mounting with Fluoroshield mounting media with DAPI (Sigma-Aldrich). Images were captured via whole-slide scanning with a Zeiss SP8 and analyzed via Fiji software.

### Codetection by indexing (CODEX) staining, imaging, processing, and analysis

Five-micron-thick slices of frozen spleens from control and MCMV-infected mice were prepared and subjected to CODEX staining as described previously [96]. Briefly, the sections were dried for 15 min and fixed for 10 min in ice-cold acetone. The samples were subsequently rehydrated in hydration buffer and photobleached. Afterwards, the sections were blocked and stained with rat anti-human/mouse B220 (RA3-6B2) (BioLegend), rat anti-mouse CD169 (3D6.112) (BioLegend), rat anti-mouse CD207/Langerin (eBioRMUL.2) (Thermo Fisher Scientific), rat anti-mouse CD49d (9C10) (BioLegend), and rabbit anti-GFP (Thermo Fisher Scientific) antibodies overnight at 4 °C. The samples were washed twice with staining buffer, fixed in ice-cold methanol, washed with 1x PBS, and fixed with BS3 fixative for 20 min (Sigma Aldrich, St. Louis, MO, USA). After removal of the fixative, the stained samples were stored at 4 °C before imaging. Then, the coverslips and reporter plate were equilibrated at room temperature for 30 minutes before imaging. A multicycle CODEX experiment was performed following the manufacturer’s instructions (Akoya Biosciences). Images were acquired via a Zeiss Axio Observer widefield fluorescence microscope with a 20x objective (NA 0.85). A z-spacing of 1.5 µm was used for acquisition. The 405, 488, 568, and 647 nm channels were implemented. The raw files were exported via CODEX Instrument Manager (Akoya Biosciences, Marlborough, MA, USA) and processed via CODEX Processor v1.7 (Akoya Biosciences). Images were inspected and analyzed in QuPath. After total cells were selected via the DAPI counterstain, multiple classifiers were established via single stains and used to gate the cells via a strategy similar to that used for flow cytometry.

### DC phagocytosis assay

CD11c^+^ DCs were isolated with CD11c MACS microbeads (Miltenyi Biotec) and diluted in medium to a concentration of 1.5 × 10^6^ cells/ml, and 100 μl of cells were plated in a 96-well round-bottom plate. OVA-AF488 at gradient concentrations diluted with PBS (0 μg/ml, 0.3 μg/ml, 1 μg/ml, 3 μg/ml, 5 μg/ml, and 10 μg/ml) was added to the DCs, which were subsequently incubated for 30 minutes at 37 °C in a cell culture incubator. The plate of an identical assay was kept on ice as a negative control. Excess fluorochrome bound to the cell surface was quenched by adding 0.1% Trypan blue stain and incubating for three minutes at room temperature. Afterwards, the cells were washed three times with PBS and then stained for flow cytometry.

### DC processing assay

CD11c^+^ DCs were isolated with CD11c MACS microbeads (Miltenyi Biotec) and diluted in medium to a concentration of 1.5 × 10^6^ cells/ml, and 100 μl of cells were plated in a 96-well round bottom plate. DQ-OVA was diluted in medium to a concentration of 2 μg/ml or 10 μg/ml. Diluted DQ-OVA was incubated with CD11c^+^ DCs in a 37 °C cell culture incubator according to the following time points: 0 (DQ-OVA was added immediately before centrifugation), 30, 60, 90, and 120 minutes. The plate of an identical assay was kept on ice as a negative control. After incubation, the cells were centrifuged and stained for flow cytometry.

### Cell isolation and coculture experiments

OVA-specific T-cell transgenic (TCR) CD8^+^ T-cell isolation was performed on the spleen and peripheral LNs of OT-I mice via the Mouse CD8a^+^ T-Cell Isolation Kit (Miltenyi Biotec) according to the manufacturer’s instructions. For the cDC1/OVA or SIINFEKL/OT-I coculture experiments, purified 4 × 10^4^ XCR1^+^ cDC1s, CD103^high^ cDC1s or CD103^-^ cDC1s were incubated with OVA or the SIINFEKL peptide for 60 min at 37 °C in a cell culture incubator; subsequently, XCR1^+^ cDC1s, CD103^high^ cDC1s or CD103^-^ cDC1s were extensively washed and cultured with 1 × 10^5^ Trace Violet (Thermo Fisher)-labeled OT-I CD8^+^ T cells. After 3 days, the proliferation of OT-I CD8^+^ T cells was analyzed via flow cytometry. In the MCMV-SIINFEKL-infected cDC1s and OT-I CD8^+^ T-cell coculture experiments, purified XCR1^+^ cDC1s, CD103^high^ cDC1s or CD103^-^ cDC1s from the spleen 18 hours after MCMV-SIINFEKL infection were cultured with trace violet (Thermo Fisher)-labeled OT-I CD8^+^ T cells at different ratios. The dilution of the cell mixture was analyzed by flow cytometry on day 3 of culture. For the MCMV-SIINFEKL-infected B-cell and OT-I CD8^+^ T-cell coculture experiments, B cells were purified 18 hours after MCMV-SIINFEKL infection in WT mice via MACS CD19 microbeads (Miltenyi Biotec) according to the manufacturer’s protocol. Purified B cells were cultured with trace violet (Thermo Fisher)-labeled OT-I CD8^+^ T cells at different ratios. After 3 days, the proliferation of OT-I CD8^+^ T cells was analyzed via flow cytometry.

### J_H_T mouse serum transfer

J_H_T mice were adoptively transferred with mouse serum derived from normal C57BL/6 mice and mouse serum derived from MCMV-infected C57BL/6 J mice 3 weeks after infection. The transfer started three days before infection, followed by one serum transfer on the day of MCMV infection and one serum transfer during the seven days of MCMV infection.

### B-cell transfer experiment

J_H_T mice were intravenously injected with 50×10^6^ MACS-purified B cells from the spleens of C57BL/6 mice (Miltenyi Biotec). One week after transfer, the footpads of the recipients were infected with 2×10^5^ PFU of MCMV. Seven days post-infection, the splenocytes were analyzed for IFN-γ expression after in vitro stimulation with the MCMV peptides.

### OT-I adoptive transfer

Mice were i.v. injected with 5 × 10^6^ cell trace violet (Thermo Fisher)-labeled MACS-purified CD8^+^ T cells from the spleen and peripheral LNs of CD45.1^+^ OT-I mice (Miltenyi Biotec). Recipients were infected by intraperitoneal (i.p.) injection of 2×10^5^ PFU of MCMV-SIINFEKL after 24 hours. Spleens were removed 3 days after infection, and the proliferation of transferred CD45.1^+^ OTI CD8^+^ T cells was analyzed via flow cytometry.

### In vivo Ag transfer assay

The mice were injected intravenously with 1 μg of αCD169-AF488 or clg-AF488 in the presence of 25 μg of poly(I:C) and 25 μg of αCD40. Two hours after the application, the mice were sacrificed, and the spleens were frozen for immunofluorescence analysis or digested for FACS analysis.

### Ex Vivo Ag Presentation Assays

The mice were injected i.v. with 1 μg of αCD169-OVA or clg-OVA plus 25 μg of αCD40 and 25 μg of poly (I:C). Sixteen hours later, purified CD103^high^ cDC1s or CD103^-^ cDC1s from the spleen were cultured with trace violet (Thermo Fisher)-labeled OT-I CD8^+^ T cells at different ratios. The dilution of the cell mixture was analyzed by flow cytometry on day 3 of culture.

### Bone marrow chimeras

To generate mice in which B cells lack LTβ expression, lethally irradiated C57BL/6 J recipients were reconstituted with 80% J_H_T bone marrow plus 20% LTβ^−/−^ bone marrow. Controls were created by reconstituting lethally irradiated C57BL/6 J recipients with 80% J_H_T bone marrow plus 20% WT bone marrow. The recipient mice received antibiotic-containing drinking water starting 10 days before being lethally irradiated, and the treatment was continued for 6 weeks following bone marrow reconstitution. Eight weeks after reconstitution, the mice were infected with MCMV and analyzed.

### VCAM1 blockade

The mice were injected i.v. with 10 mg/kg anti-VCAM1 mAb (clone M/K 2.7) (Bio X Cell) or rat IgG1 isotype control (clone HRPN) (Bio X Cell) every 3 days for 1 week.

### VLA-4 blockade

Animals were treated with i.v. anti-VLA-4 mAb (clone PS/2) (Bio-XCell) or 200 µg isotype control antibody every 3 days for 1 week.

### AAV design and applications

For knockdown of VCAM1 specifically in macrophages, AAV-CD68 was used to deliver short hairpin (sh) RNA against VCAM1 to the target cells. The AAV-CD68-shRNA-VCAM1 and control AAV-CD68-shRNA-NC constructs were produced by PackGene, Switzerland. The AAVs were delivered as high-titer stocks that were ready to use.

### In situ proximity ligation assay

For the in situ proximity ligation assay (PLA), we used the Duolink® In Situ Red Starter Kit Mouse/Rabbit (Sigma‒Aldrich, DUO92101-1KT) according to the manufacturer’s recommendation with the solutions supplied in the kit. Whole spleens were cut into 6 mm sections with a cryostat, fixed with 4% PFA in PBS for 10 min at 37 °C, and permeabilized with 0.2% Triton X-100 for 10 min. Slides were blocked with blocking solution for 1 hour at 37 °C and subsequently incubated with a rabbit polyclonal anti-Itga4 antibody (clone JA09-36) (Thermo Fisher) and a mouse monoclonal anti-Vcam1 antibody (clone 1.4C3) (Thermo Fisher) at 4 °C overnight. After PLA probe incubation, ligation, amplification, and washing, the slides were mounted with Duolink® Mounting Medium containing DAPI and sealed with nail polish. The samples were stored in the dark at 4 °C until imaging. Images were captured via whole-slide scanning with a Zeiss SP8 and analyzed via Fiji software.

### RNA isolation and bulk RNA sequencing

RNA was purified from MCMV-M45 tetramer^+^CD8^+^ T cells with the RNeasy Plus Micro Kit according to the manufacturer’s protocol (Qiagen). The RNA was quantified with a Qubit flex fluorometer (Invitrogen), and the quality was assessed on a Bioanalyzer 2100 (Agilent) via an RNA 6000 Pico chip (Agilent). Samples with an RNA integrity number (RIN) of > 8 were used for cDNA amplification via the Smart-Seq V4 Ultra Low Input RNA Kit (Takara). Libraries were made from 1 ng of full-length cDNA via the Nextera-XT DNA Sample Preparation Kit (Illumina) according to the manufacturer’s instructions. The quantity of the cDNA libraries was assessed with a Qubit flex fluorometer (Invitrogen), and the average library size was determined via Agilent’s 2100 Bioanalyzer HS DNA assay. The quantified libraries were sent to Novogene (Cambridge, UK) and sequenced on a NovaSeq 6000 (Illumina) to generate approximately 30 million PE150 reads for each sample.

### RNA sequencing analysis

The sequence reads were trimmed for adaptor sequences before being analyzed with Qiagen’s CLC Genomics Workbench (v23.0.2 with CLC’s default settings for RNA-Seq analysis). The raw RNA sequencing reads were mapped to the reference genome GRCm38.

### Single-cell RNA sequencing

Splenic CD11c^+^ cells were enriched with CD11c-MACS beads (Miltenyi Biotec), and then the cells were labeled with the Mouse Immune Single-Cell Multiplexing Kit (BD Biosciences). The labeled cells were counted and captured via the BD Rhapsody Single-Cell Analysis System Instrument (BD Biosciences) according to the provided instructions. Subsequently, whole-transcriptome analysis (WTA) library preparation and sample tag library preparation were performed following the BD Rhapsody System mRNA WTA and sample tag library preparation protocol. The quality and quantity of the cDNA libraries were checked via the Agilent Bioanalyzer 2100 (Agilent), and sequencing (150 PE reads aiming for 50,000 raw reads/cell) was performed at Novogene (Cambridge, UK) via the Illumina NovaSeq 6000 (Illumina). The generated raw data were preprocessed according to the Illumina standard protocol, and transcript alignment, counting, and demultiplexing were carried out via the BD Rhapsody WTA analytic pipeline. The output matrices RSEC_MolsPerCell were further read into R (version 4.2.3) separately and converted to Seurat objects via the Seurat R package [97] (version 4.3.0.1). Genes with low expression levels (detected in fewer than 3 cells) and cells that contained fewer than 200 detected genes were excluded from the analysis. Cells that did not meet the threshold of the mean ± 3 times the median absolute deviation of the number of mitochondrial genes, the total number of genes, and the number of transcripts detected per cell for each separate sample were filtered out. The following processing and parameters were subsequently applied: normalization, variable feature search and scaling via the transform, data integration, dimensional reduction via PCA and UMAP embedding (using 30 PCA dimensions), finding neighbors (using 30 PCA dimensions), and cluster search (resolution = 0.7). For each cluster, a main cell type was manually identified on the basis of classical marker gene expression. The cluster identified as cDC1s was subjected to subsetting via the “subset” function. Cell‒cell communication analysis was performed via the CellChat R package. MMMs and Langerin^+^ cDC1s were identified and subset from the mouse dataset via Seurat. Specifically, MMMs were defined as cells with Siglec1 expression > 0.1, whereas Langerin^+^ cDC1s were defined as cells with CD207 expression > 0.1. These subsets were then used to create a CellChat object for downstream analysis on the basis of standardized procedures as previously described [98, 99]. The inherent mouse database encompassing “secreted signaling”, “ECM-receptor” and “cell‒cell contact” types of communication was employed to infer intercellular communication networks on the basis of known ligand‒receptor interactions, facilitating the identification of key signaling pathways and interactions between these cell populations. Cell groups with fewer than 5 cells were filtered out via the filter communication function in CellChat.

### Statistical analysis

The data from all the experiments are presented as the means ± SEMs. Statistical analyses were performed with two-tailed, unpaired Student’s t tests to compare two groups or one-way ANOVA to compare multiple groups. Sample sizes were determined by experimental complexity, but no methods were used to determine the normal distribution of the samples. Statistical analyses were performed via GraphPad Prism (version 9.4.0). A *P* value ≤ 0.05 was considered significant.

## Supplementary information

Supplementary Figure 1

Supplementary Figure 2

Supplementary Figure 3

Supplementary Figure 4

Supplementary Figure 5

Supplementary Figure 6

Supplementary Figure 7

Supplementary information

## Data Availability

All RNA-sequencing data described in the paper have been deposited in the NCBI Gene Expression Omnibus (GEO) under accession code GSE315617 (bulk RNA-seq) and GSE315452 (scRNA-seq). All other data needed to evaluate the conclusions in the paper are present in the paper or the Supplementary Materials.
