# Supplementary figures and images for: B cells maintain the homeostasis of splenic marginal zone antigen-presenting cells to promote the antiviral CD8+ T-cell response
Source: Cell Mol Immunol. 2026 Feb 24;23(4):383–99. doi: 10.1038/s41423-026-01392-0 (PMC13035995; doi:10.1038/s41423-026-01392-0)

**A**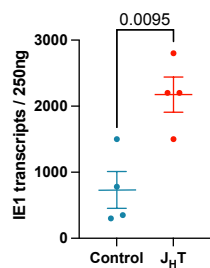**B**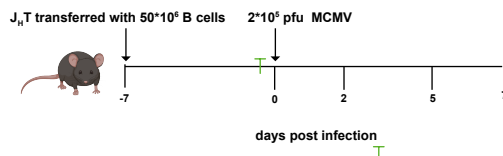**C**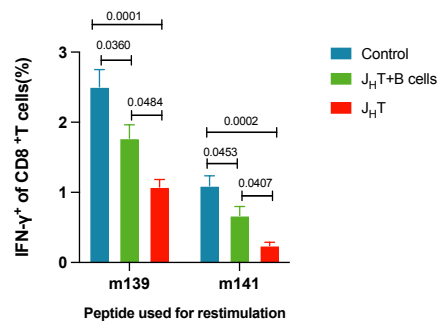**D**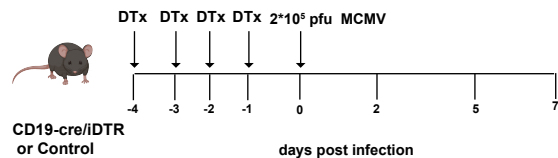**E**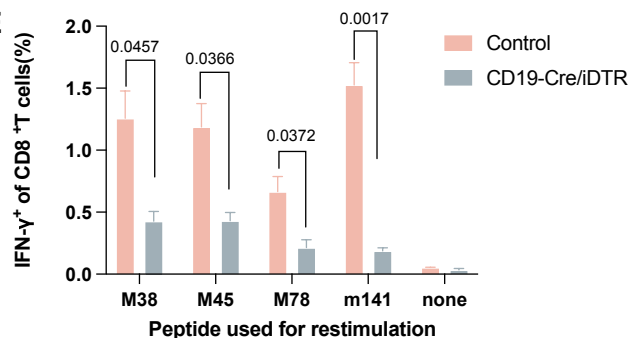**F**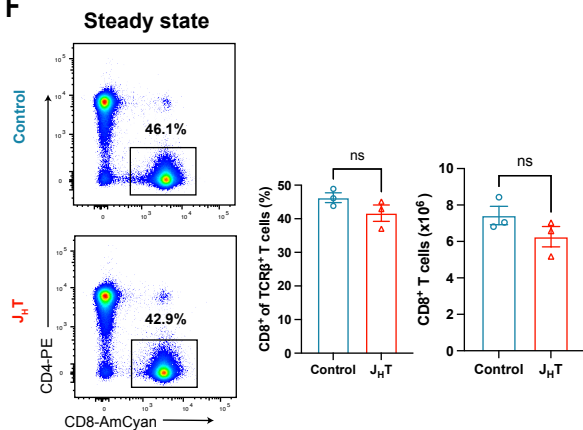**G**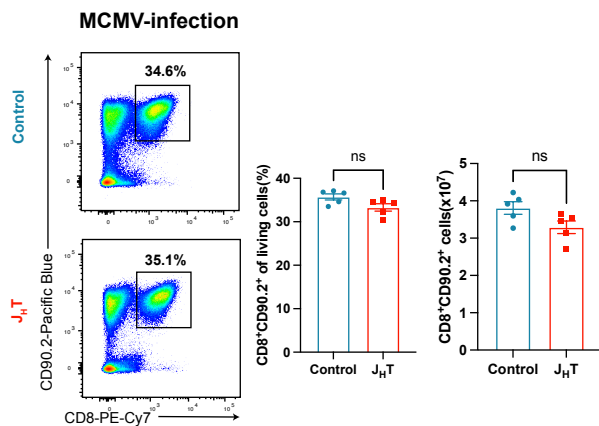

Supplement: Supplementary file 1 — Supplementary Figure 1 [file 41423_2026_1392_MOESM1_ESM.pdf]

**A**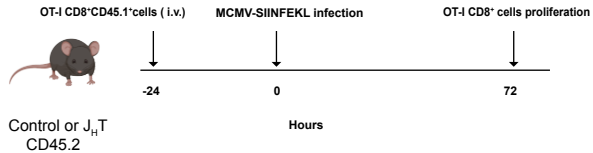**B****Gated on TCR $\beta$ <sup>+</sup>CD19<sup>+</sup>living cells**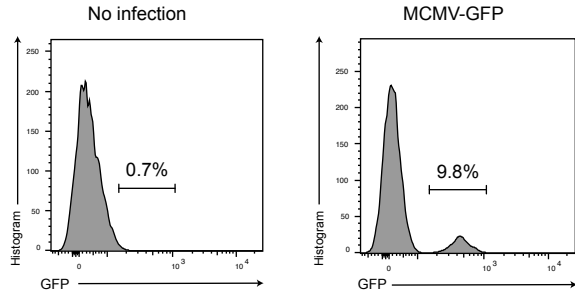

Supplement: Supplementary file 2 — Supplementary Figure 2 [file 41423_2026_1392_MOESM2_ESM.pdf]

**A**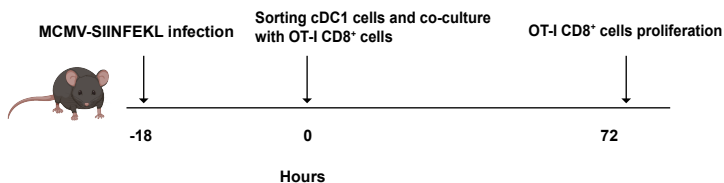**B**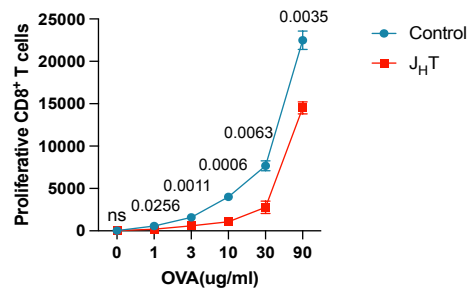**C**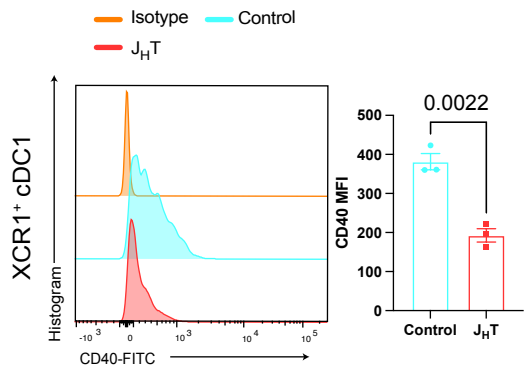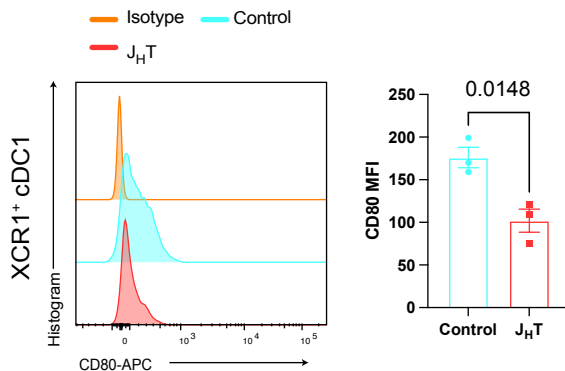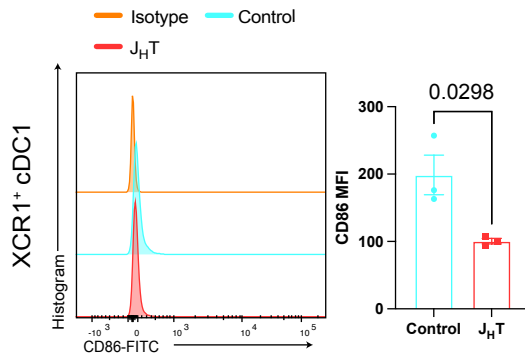**D**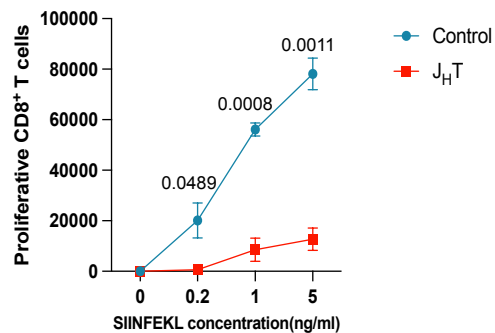

Supplement: Supplementary file 3 — Supplementary Figure 3 [file 41423_2026_1392_MOESM3_ESM.pdf]

**A**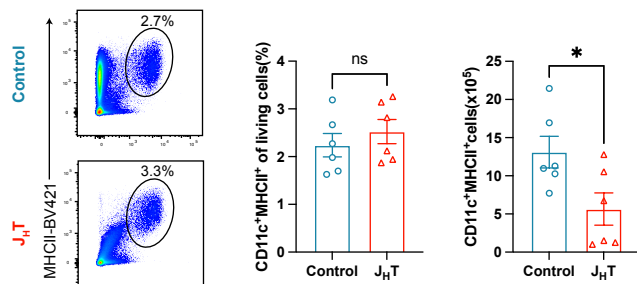**B**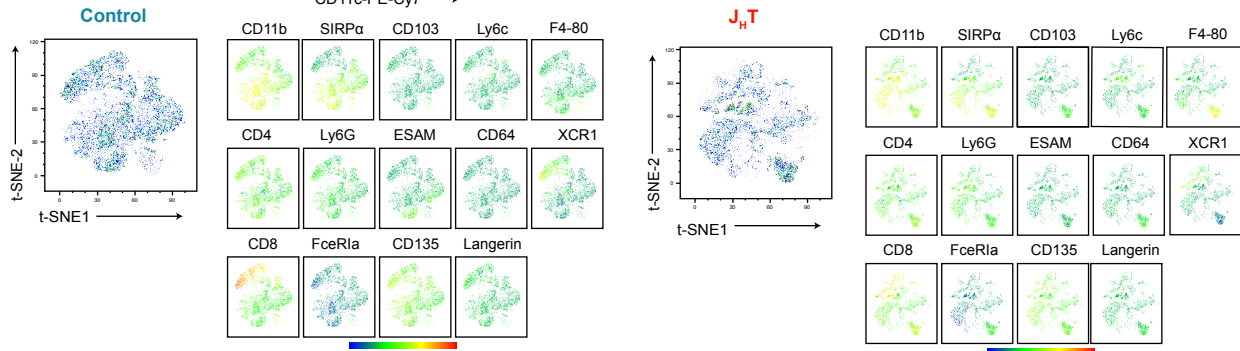**C**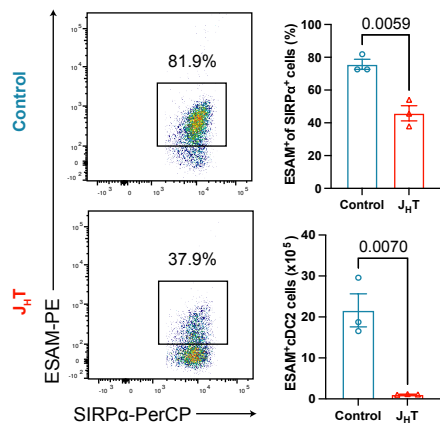**D**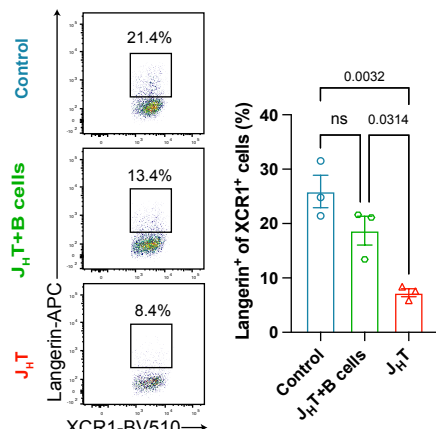**E**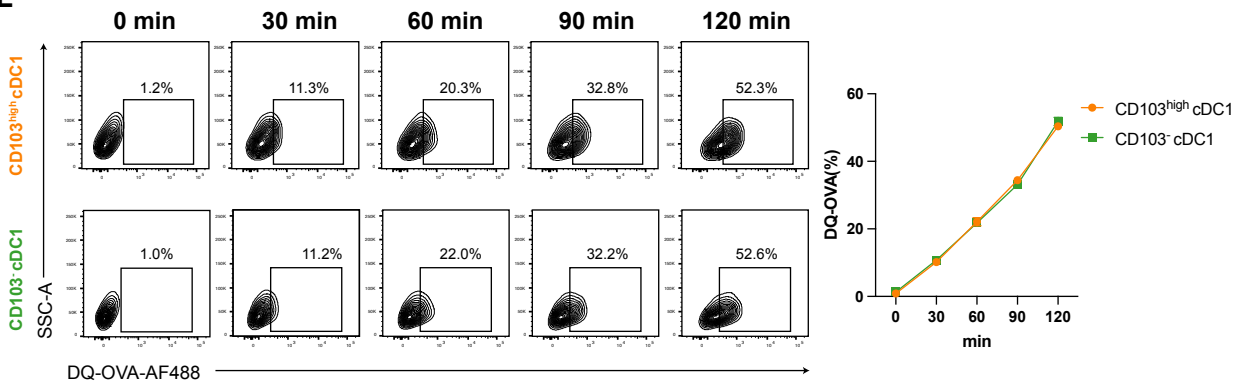**F**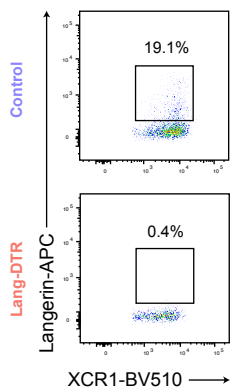**G**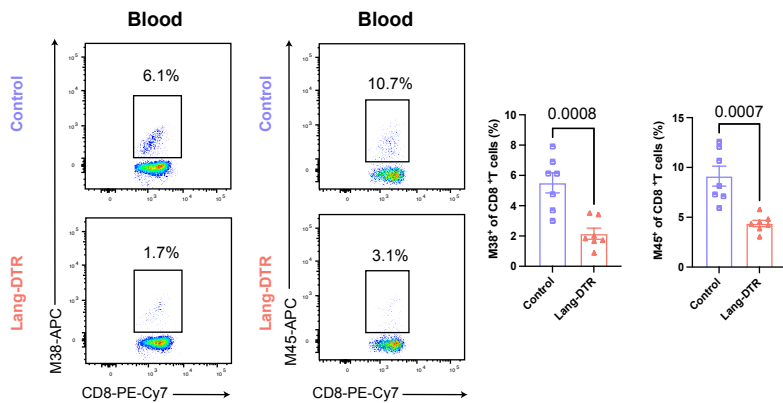

Supplement: Supplementary file 4 — Supplementary Figure 4 [file 41423_2026_1392_MOESM4_ESM.pdf]

**A**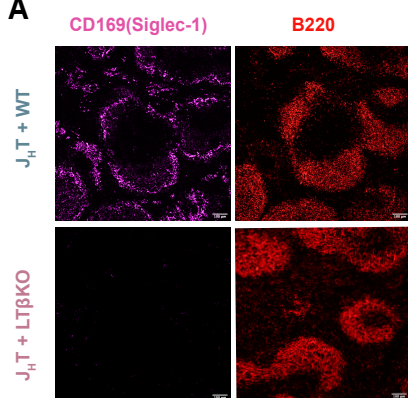**B**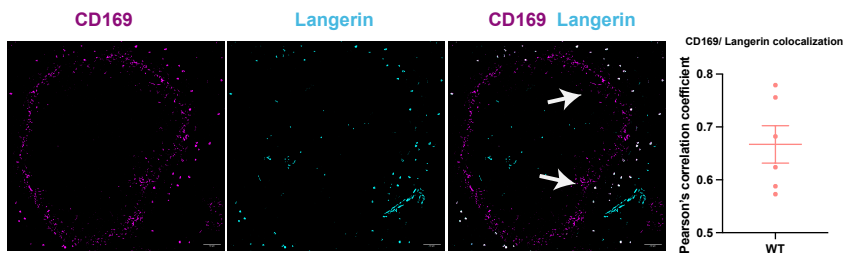**C**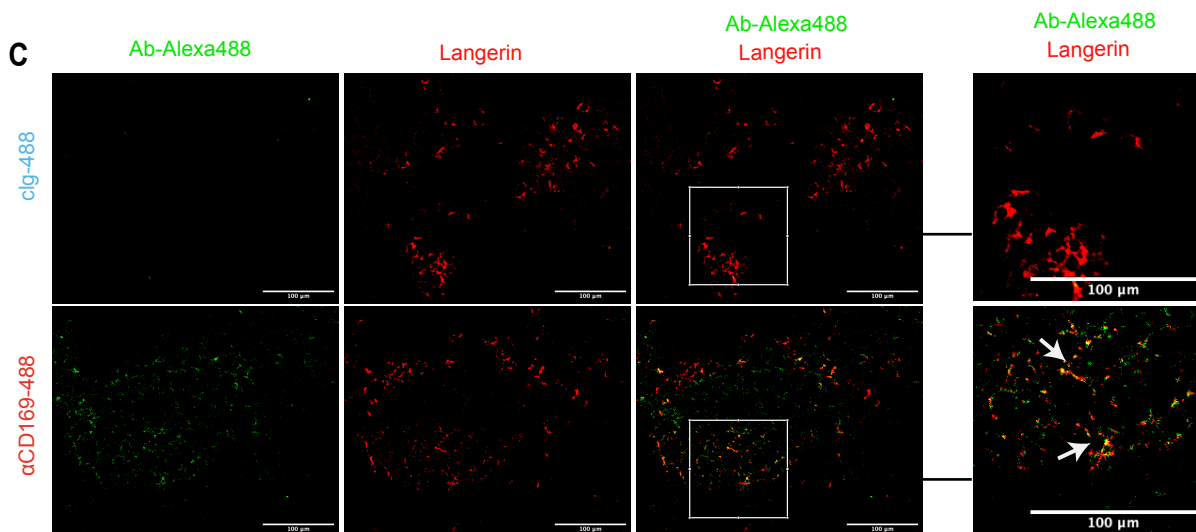**D**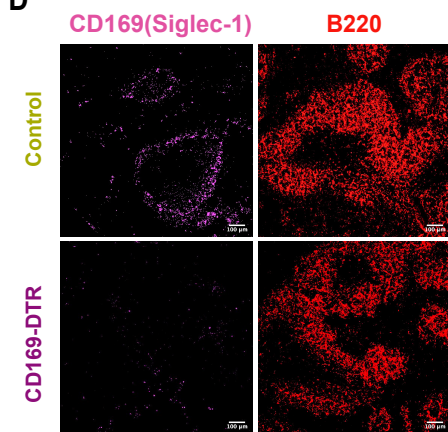**E**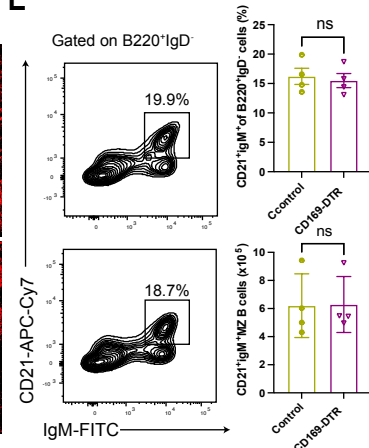**F**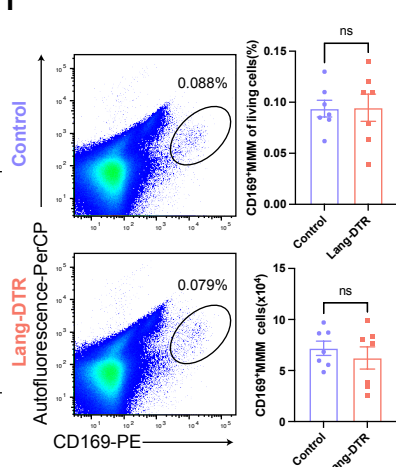**G**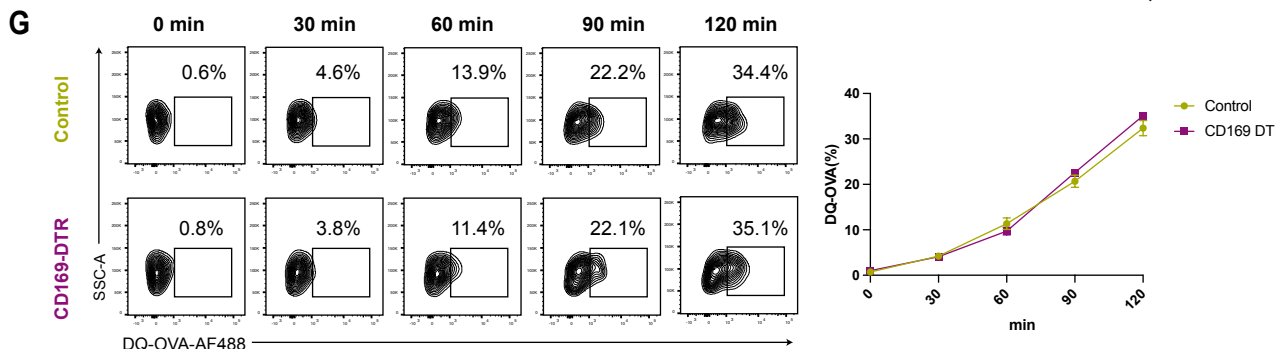**H**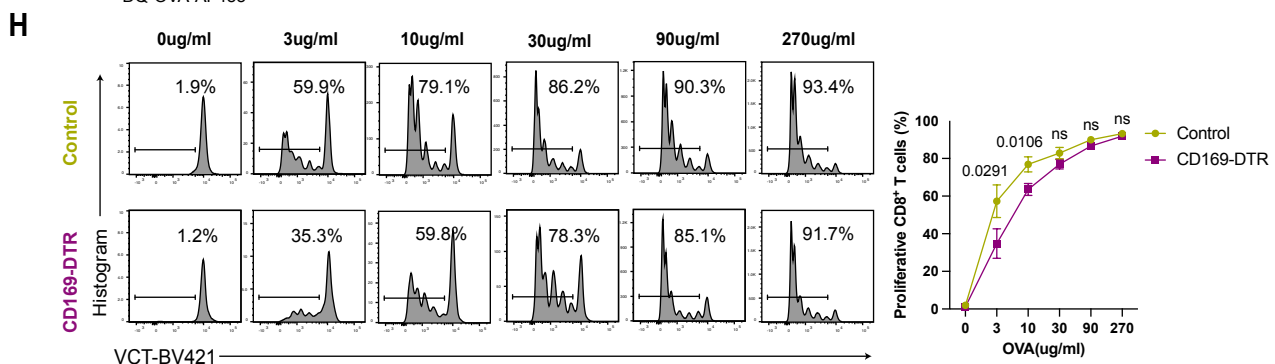

Supplement: Supplementary file 5 — Supplementary Figure 5 [file 41423_2026_1392_MOESM5_ESM.pdf]

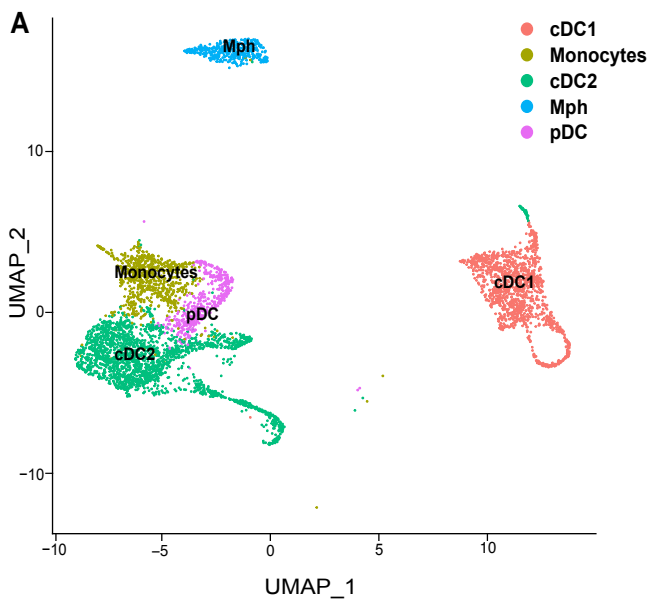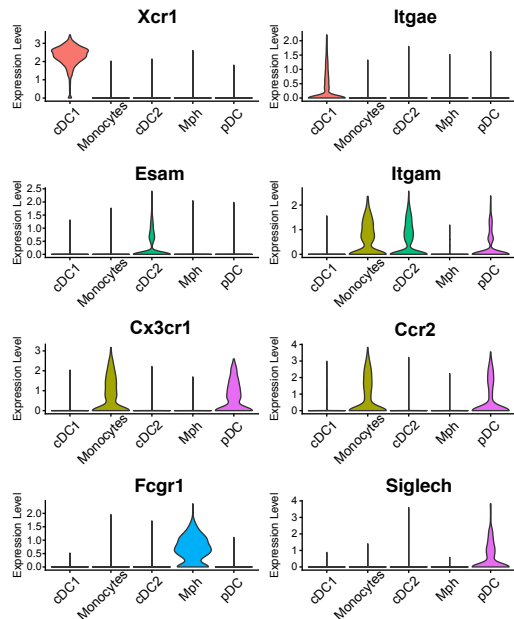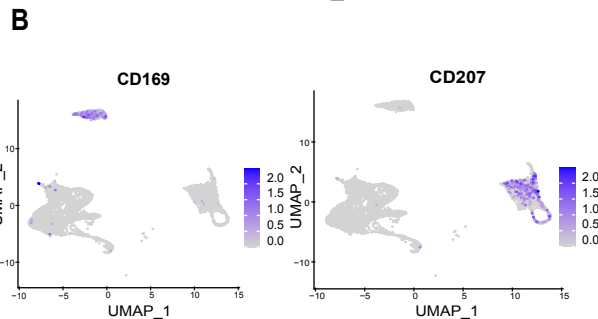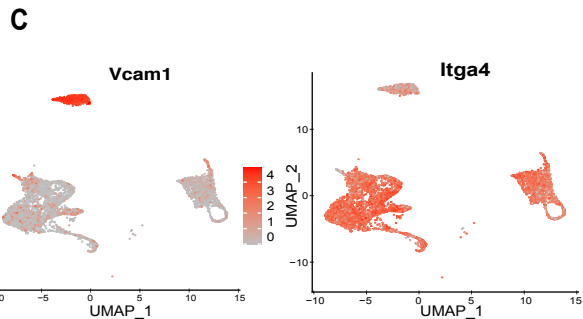

Supplement: Supplementary file 6 — Supplementary Figure 6 [file 41423_2026_1392_MOESM6_ESM.pdf]

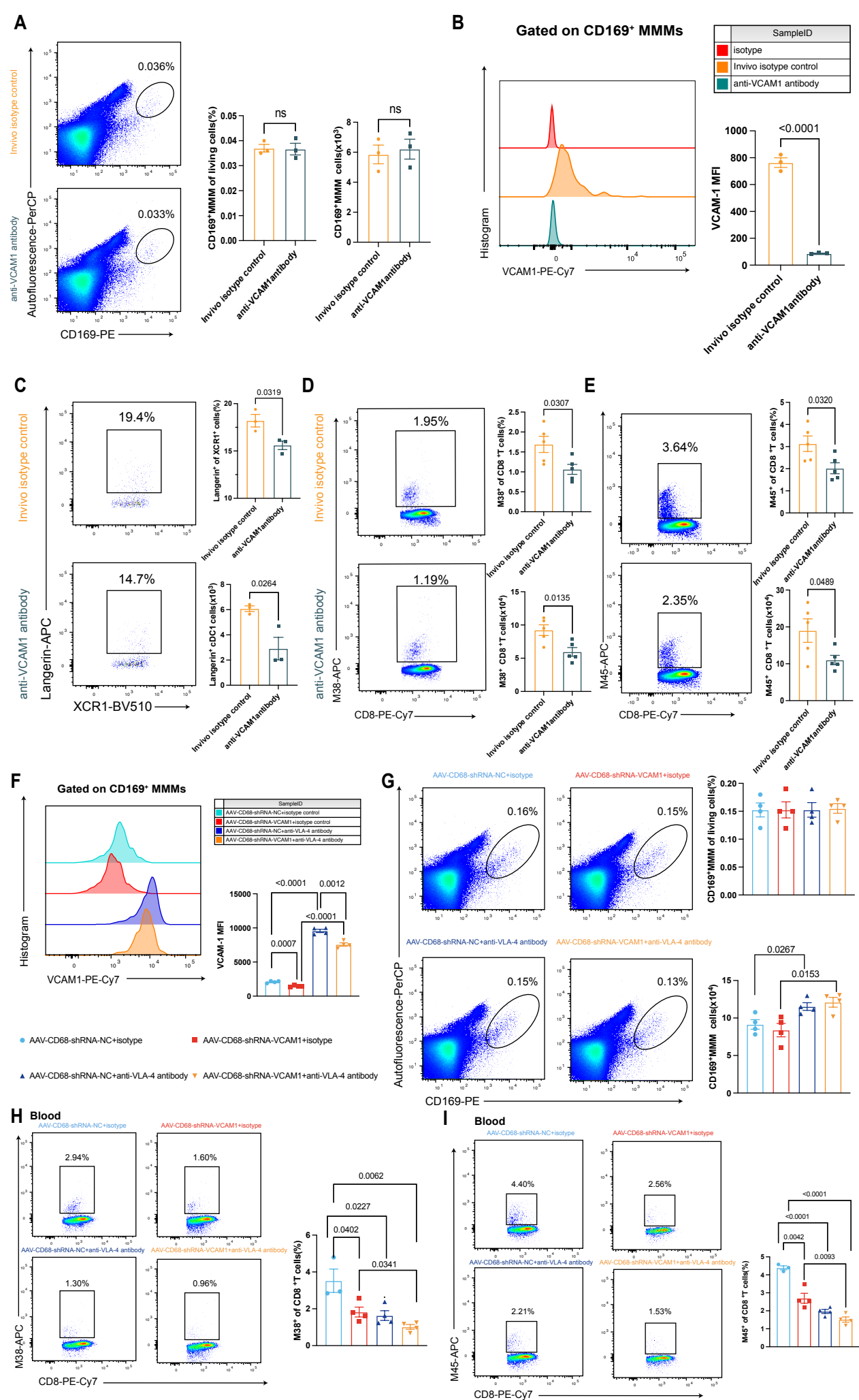

Supplement: Supplementary file 7 — Supplementary Figure 7 [file 41423_2026_1392_MOESM7_ESM.pdf]
