# Supplementary material for: B cells maintain the homeostasis of splenic marginal zone antigen-presenting cells to promote the antiviral CD8+ T-cell response
Source: Cell Mol Immunol. 2026 Feb 24;23(4):383–99. doi: 10.1038/s41423-026-01392-0 (PMC13035995; doi:10.1038/s41423-026-01392-0)
Supplement: Supplementary file 8 — Supplementary information [file 41423_2026_1392_MOESM8_ESM.docx]

**Supplementary Figure Legends**:

**Fig. S1. B cells are essential for the maintenance of the primary MCMV-specific CD8^+^ T-cell response.**

(**A**) IE1 transcript levels in the popliteal lymph nodes at 2 days post-MCMV infection were measured via qRT‒PCR (n = 4). (**B**) Schematic diagram outlining the program for the reconstitution of J_H_T mice with WT splenic B cells; 1 week after B-cell transfer, the mice were infected with MCMV. (**C**) Frequencies of IFN-γ-producing CD8^+^ T cells in the spleen after stimulation with different dominant MCMV peptides at 7 days post-infection in control, J_H_T, or B-cell-reconstituted J_H_T mice (n = 7). (**D**) Schematic diagram outlining the program for CD19-Cre/iDTR and iDTR mice treated with DTx according to the illustrated schedule; then, the mice were infected with MCMV. (**E**) Frequency of IFN-γ-producing CD8^+^ T cells in the spleen of control or CD19-Cre/iDTR mice 7 days after stimulation with MCMV peptides (n =3-7). (**F**) Representative flow cytometry dot plots of splenic CD8+ T cells from control or J_H_T-treated mice at steady state. The bar graphs show the frequencies (left) and absolute numbers (right) of CD8+ T cells in each group (n = 3). (**G**) Representative flow cytometry dot plots of splenic CD90.2^+^ CD8+ T cells in MCMV-infected control or J_H_T mice. The bar graphs show the frequencies (left) and absolute numbers (right) of CD8+ T cells in each group (n = 5). The data are presented as the means ± SEMs and are representative of two to three independent experiments. Statistical analysis: Student’s t test, Fig. S1A, 1E, 1F, 1G. One-way ANOVA, Fig. S1C.

**Fig. S2.** **Loss of B cells reduces the priming of CD8^+^ T cells after viral infection**

(**A**) Schematic diagram outlining the program for OTI CD8^+^ T-cell transfer experiments. Twenty-four hours after OTI CD8^+^ T-cell transfer, the mice were infected with MCMV-SIINFEKL. (**B**) Flow cytometry histograms of MCMV-EGFP^+^ B cells in the spleens of MCMV-EGFP-infected WT mice and uninfected control WT mice.

**Fig. S3. B cells** **contribute XCR1+CD8+ cDC1s to prime CD8^+^ T cells.**

(**A**) Schematic diagram outlining the program used to coculture OVA-specific transgenic OTI CD8^+^ T cells with cDC1s isolated from MCMV-SIINFEKL-infected control or J_H_T mice. (**B**) Line graph indicating the absolute numbers of proliferating OTI CD8+ T cells cocultured with cDC1s isolated from control or J_H_T mice and pulsed with OVA protein (n = 5). (**C**) Flow cytometry histograms showing the expression of CD40 (upper left), CD80 (upper right), and CD86 (bottom left) in cDC1s from control or J_H_T mice. The bar graphs show the median fluorescence intensity (MFI) of CD40 (upper left), CD80 (upper right), and CD86 (bottom left) in cDC1s from control or J_H_T mice (n = 3). (**D**) Line graph indicating the absolute numbers of proliferating OTI CD8+ T cells cocultured with cDC1s isolated from control or J_H_T mice and pulsed with the SIINFEKL peptide (n = 6). The data are presented as the means ± SEMs and are representative of two to three independent experiments. Statistical analysis: Student’s t test, Fig. S3B- S3D.

**Fig. S4. The homeostasis of splenic Langerin^+^ cDC1s is regulated by B cells.**

(**A**) Flow cytometry dot plots of CD11c^+^MHCII^+^ cDCs in the spleens of control or J_H_T mice. The bar graphs indicate the frequencies (left) and absolute numbers (right) of CD11c^+^MHCII^+^ cDCs (n = 6). (**B**) t-SNE of the expression of selected markers associated with the cDC subgroup in the control (left) and J_H_T (right) cDC populations (n = 3). (**C**) Flow cytometry plot showing splenic ESAM^+^ cDC2 cells in control or J_H_T-treated mice. The bar graphs show the frequencies (top) and absolute numbers (bottom) of ESAM^+^ cDC2s (n = 3). (**D**) Representative flow cytometry plots of splenic Langerin^+^ cDC1s in control, J_H_T, or B-cell-reconstituted J_H_T mice. The bar graphs show the frequencies of Langerin^+^ cDC1s (n = 3). (**E**) Flow cytometry plots of DQ-OVA^+^XCR1^+^ cDC1s exposed to DQ-OVA for different durations. Briefly, CD103^high^ cDC1s or CD103^-^ cDC1s isolated from WT mice were incubated with 5 μg/ml DQ-OVA for 0, 30, 60, 90, or 120 minutes at 37°C and 5% CO_2_. The line graph shows the frequencies of DQ-OVA^+^XCR1^+^ cDC1s (n = 8). (**F**) Flow cytometry plot of splenic Langerin^+^ cDC1s in control and Lang-DTR mice after diphtheria toxin treatment. (**G**) Flow cytometry plot of MCMV-M38 (left) or MCMV-M45 (right) tetramer^+^ CD8^+^ T cells in the blood of control or Lang-DTR mice. The bar graphs show the frequencies of MCMV-M38 (left) or MCMV-M45 (right) tetramer^+^CD8^+^ T cells (n = 7). The data are presented as the means ± SEMs and are representative of five (S4A and S4C) or two (S4B, S4D-S4G) independent experiments. Statistical analysis: Student’s t test; Fig. S4A, S4C, S4E, and S4G. One-way ANOVA, Fig. S4D.

**Fig. S5. B cells expressing LTβ maintain MMMs to support the homeostasis of splenic Langerin^+^ cDC1s**

(**A**) Immunofluorescence images of CD169 (Siglec-1) and B220 in the spleens of J_H_T-mixed WT BM chimeric mice and J_H_T-mixed LTβ-deficient BM chimeric mice (scale bar, 100 µm; n = 3). (**B**) Immunofluorescence images of CD169 (Siglec-1) and Langerin in the spleens of WT mice; the data are presented as Pearson’s correlation coefficient (PCC) values (scale bar, 50 µm; n = 3). (**C**) Immunofluorescence images of the spleens of WT mice injected with Ab-Alexa488 and Langerin (scale bar, 100 µm; n = 4). (**D**) Immunofluorescence images of CD169 (Siglec-1) and B220 in the spleens of control and CD169-DTR mice (scale bar, 100 µm; n = 4). (**E**) Flow cytometry contour plot of IgD^-^CD21^+^IgM^+^ MZ B cells in the spleens of control or CD169-DTR mice. The bar graphs show the frequencies (top) and absolute numbers (bottom) of MZ B cells (n = 4). (**F**) Flow cytometry dot plots showing MMMs in the spleens of control and Lang-DTR mice. MMMs were identified as live CD45^+^ autofluorescent CD169^+^ cells. The bar graphs indicate the frequencies (top) and absolute numbers (bottom) of MMMs (n = 6). (**G**) Flow cytometry plots of DQ-OVA^+^XCR1^+^ cDC1s exposed to DQ-OVA for different durations. Briefly, cDC1s isolated from control or CD169-DTR mice were incubated with 2 μg/ml DQ-OVA for 0, 30, 60, 90, or 120 minutes at 37°C and 5% CO_2_. The line graph shows the frequencies of DQ-OVA ^+^XCR1^+^ cDC1s (n = 5). (**H**) Proliferation of OVA-specific transgenic OTI CD8^+^ T cells cocultured with cDC1s isolated from control or CD169-DTR mice and pulsed with OVA protein. Line graphs indicate the frequencies of proliferating OTI CD8^+^ T cells (n = 5). The data are presented as the means ± SEMs and are representative of two independent experiments. Statistical analysis: Student’s t test; Fig. S5E- S5H.

**Fig. S6. Single-cell profiling identifies key communication axes between MMMs and Langerin⁺XCR1⁺ cDC1s**

(**A**) UMAP plots of the indicated clusters (left) and violin plots of their feature genes (right). (**B**) UMAP plots showing MMMs and Langerin^+^ cDC1 clusters. (**C**) UMAP plots showing VCAM1 and ITGA4 expression in the indicated clusters.

**Fig. S7. MMMs via VCAM1-ITGA4/ITGB1 cross-talk with Langerin+XCR1+ cDC1s**

(**A**) Representative flow cytometry dot plots of MMMs in the spleens of control IgG- or anti-VCAM1 antibody-treated mice. The bar graphs indicate the frequencies (left) and absolute numbers (right) of MMMs (n = 3). (**B**) Flow cytometry histograms showing the expression of VCAM1 in MMMs from control IgG- or anti-VCAM1 antibody-treated mice. The bar graphs show the median fluorescence intensity (MFI) of VCAM1 in control IgG- or anti-VCAM1 antibody-treated mice (n = 3). (**C)** Representative flow cytometry dot plots of splenic Langerin^+^ cDC1s from control IgG- or anti-VCAM1 antibody-treated mice. The bar graphs show the frequencies (top) and absolute numbers (bottom) of Langerin^+^ cDC1s (n = 3). (**D)** Representative flow cytometry dot plots of splenic MCMV-M38 tetramer^+^CD8^+^ T cells in control IgG- or anti-VCAM1 antibody-treated mice. The bar graphs show the frequencies (top) and absolute numbers (bottom) of MCMV-M38 tetramer^+^CD8^+^ T cells in each group (n = 5). (**E**) Flow cytometry dot plot showing splenic MCMV-M45 tetramer^+^CD8^+^ T cells in control IgG- or anti-VCAM1 antibody-treated mice. The bar graphs show the frequencies (top) and absolute numbers (bottom) of MCMV-M45 tetramer^+^CD8^+^ T cells in each group (n = 5). (**F**) Flow cytometry histograms showing the expression of VCAM1 in MMMs from AAV-CD68-shRNA-NC+isotype, AAV-CD68-shRNA-VCAM1+isotype, AAV-CD68-shRNA-NC+anti-VLA-4 antibody-, and AAV-CD68-shRNA-VCAM1+anti-VLA-4 antibody-injected mice. The bar graphs show the median fluorescence intensity (MFI) of VCAM1 (n = 4). (**G**) Representative flow cytometry dot plots of MMMs in the spleens of AAV-CD68-shRNA-NC+isotype (upper left), AAV-CD68-shRNA-VCAM1+isotype (upper right), AAV-CD68-shRNA-NC+anti-VLA-4 antibody (bottom left), and AAV-CD68-shRNA-VCAM1+anti-VLA-4 antibody (bottom right)-injected mice. The bar graphs indicate the frequencies (top) and absolute numbers (bottom) of MMMs (n = 4). (**H**) Representative flow cytometry dot plots of MCMV-M38 tetramer^+^CD8^+^ T cells in the blood from AAV-CD68-shRNA-NC+isotype (upper left), AAV-CD68-shRNA-VCAM1+isotype (upper right), AAV-CD68-shRNA-NC+anti-VLA-4 antibody (bottom left), and AAV-CD68-shRNA-VCAM1+anti-VLA-4 antibody (bottom right)-injected mice. The bar graphs show the frequencies of MCMV-M38 tetramer^+^CD8^+^ T cells in each group (n = 3--4). (**I**) Flow cytometry dot plot showing MCMV-M45 tetramer^+^CD8^+^ T cells in the blood of AAV-CD68-shRNA-NC+isotype (upper left), AAV-CD68-shRNA-VCAM1+isotype (upper right), AAV-CD68-shRNA-NC+anti-VLA-4 antibody (bottom left), and AAV-CD68-shRNA-VCAM1+anti-VLA-4 antibody (bottom right)-injected mice. The bar graphs show the frequencies of MCMV-M45 tetramer^+^CD8^+^ T cells in each group (n = 3--4). The data are presented as the means ± SEMs. Statistical analysis: Student’s t test, Fig. S6 A-E. One-way ANOVA, Fig. S4 F-I.
